# The colorectal cancer-associated faecal microbiome of developing countries resembles that of developed countries

**DOI:** 10.1186/s13073-021-00844-8

**Published:** 2021-02-16

**Authors:** Caroline Young, Henry M. Wood, Ramakrishnan Ayloor Seshadri, Pham Van Nang, Carlos Vaccaro, Luis Contreras Melendez, Mayilvahanan Bose, Mai Van Doi, Tamara Alejandra Piñero, Camilo Tapia Valladares, Julieta Arguero, Alba Fuentes Balaguer, Kelsey N. Thompson, Yan Yan, Curtis Huttenhower, Philip Quirke

**Affiliations:** 1 Pathology & Data Analytics, Leeds Institute of Medical Research at St James’s University Hospital, University of Leeds, Level 4 Wellcome Trust Brenner Building, Leeds, LS9 7TF UK; 2 Cancer Institute (WIA), Chennai, India; 3 Can Tho University of Medicine and Pharmacy, Can Tho, Vietnam; 4 Instituto de Medicina Traslacional e Ingeniería Biomédica (IMTIB) - CONICET - Instituto Universitario del Hospital Italiano (IUHI), Hospital Italiano de buenos Aires (HIBA), Buenos Aires, Argentina; 5 Universidad de los Andes, Santiago, Chile; 6 Department of Biostatistics, Harvard T.H. Chan School of Public Health, Harvard University, Boston, USA

**Keywords:** Microbiota, gFOBT, Argentina, Chile, India, Vietnam

## Abstract

**Background:**

The incidence of colorectal cancer (CRC) is increasing in developing countries, yet limited research on the CRC- associated microbiota has been conducted in these areas, in part due to scarce resources, facilities, and the difficulty of fresh or frozen stool storage/transport. Here, we aimed (1) to establish a broad representation of diverse developing countries (Argentina, Chile, India, and Vietnam); (2) to validate a ‘resource-light’ sample-collection protocol translatable in these settings using guaiac faecal occult blood test (gFOBT) cards stored and, importantly, shipped internationally at room temperature; (3) to perform initial profiling of the collective CRC-associated microbiome of these developing countries; and (4) to compare this quantitatively with established CRC biomarkers from developed countries.

**Methods:**

We assessed the effect of international storage and transport at room temperature by replicating gFOBT from five UK volunteers, storing two in the UK, and sending replicates to institutes in the four countries. Next, to determine the effect of prolonged UK storage, DNA extraction replicates for a subset of samples were performed up to 252 days apart. To profile the CRC-associated microbiome of developing countries, gFOBT were collected from 41 treatment-naïve CRC patients and 40 non-CRC controls from across the four institutes, and V4 16S rRNA gene sequencing was performed. Finally, we constructed a random forest (RF) model that was trained and tested against existing datasets from developed countries.

**Results:**

The microbiome was stably assayed when samples were stored/transported at room temperature and after prolonged UK storage. Large-scale microbiome structure was separated by country and continent, with a smaller effect from CRC. Importantly, the RF model performed similarly to models trained using external datasets and identified similar taxa of importance (*Parvimonas*, *Peptostreptococcus*, *Fusobacterium*, *Alistipes*, and *Escherichia*).

**Conclusions:**

This study demonstrates that gFOBT, stored and transported at room temperature, represents a suitable method of faecal sample collection for amplicon-based microbiome biomarkers in developing countries and suggests a CRC-faecal microbiome association that is consistent between developed and developing countries.

**Supplementary Information:**

The online version contains supplementary material available at 10.1186/s13073-021-00844-8.

## Background

Colorectal cancer (CRC) is the fourth commonest cause of global cancer-related deaths. Incidence rates have traditionally been highest in developed countries, but are increasing in developing countries, many of which are ill-equipped to respond to this new burden of disease [1]. There is growing evidence of an association between CRC and an altered faecal microbiome, with the potential to develop novel screening, prognostic or therapeutic markers. Certain bacteria have been proposed as putative oncomicrobes and specific genetic elements such as toxins have been implicated [2]. However, the majority of CRC-microbiome research has profiled developed cohorts. Of the limited number of studies conducted in developing countries, most have been small pilot studies [3–7]. It cannot be assumed that results from developed countries will be generalisable to developing populations, as the health-associated microbiome of developed and developing populations has been shown to differ taxonomically and functionally [8].

One of the biggest limitations to conducting microbiome research in developing countries is storage and transport of frozen stool, which is widely considered the gold standard. Alternative methods have been proposed, and include storing faeces on screening cards (Flinders Technology Associates (FTA) cards or guaiac faecal occult blood test (gFOBT) CRC screening cards) at room temperature [9–15]. Two studies have indicated that cards could be used to store stool at high ambient temperatures, such as those of many developing countries [16, 17]. However, these studies did not assess the effect of international transport on microbiome stability. Many microbiome studies use samples collected on site or transported frozen, severely limiting protocol options in developing countries. Other protocols have been explored for collection, storage, and transport at ambient temperatures for gut microbiome studies in developing nations, including gFOBT card variants. However, this still requires thorough testing and optimisation for clinical use, particularly as this is not the equivalent of storage in temperature-controlled settings (transport temperatures are likely to be highly variable, encompassing transport at outside-temperature to the airport, transport within the cargo of an aeroplane and transit times of ~weeks).

To improve the field’s ability to conduct low-cost gut microbiome profiling for CRC screening in developing countries, and to provide a pilot assessment of the global CRC-linked microbiome, we established a network comprising researchers from the continents of South America (Argentina and Chile, Development Assistance Committee (DAC)-listed upper-middle income countries at the time of the study), South East Asia (India and Vietnam, DAC-listed lower-middle income countries), and Europe (UK) as a control. These countries represent a range of increasing CRC incidence rates (Age Standardised World Rate in 2018 per 100,000 person-years: India 4.4; Vietnam 13.4; Chile 20.7; Argentina 25.0; UK 32.1) [18–22]. With the exception of the UK, limited microbiome research, in particular CRC-microbiome profiling, has been conducted in these countries [6, 23, 24] We sought to address these current limitations by assessing whether gFOBT cards collected both in the country of interest and from UK volunteers, then stored/transported at ambient temperatures, could assess CRC-associated microbes comparably around the globe. After establishing the efficacy of our methodology, we compared the faecal microbiomes of ten CRC patients and ten non-CRC controls each from India, Vietnam, Chile, and Argentina, using a standardised methodology to mitigate technical biases, and found the resulting CRC-associated amplicon profiles to be comparable with those from existing CRC-associated metagenomes from developed countries.

## Methods

### UK volunteer samples

**Fig. 1:**
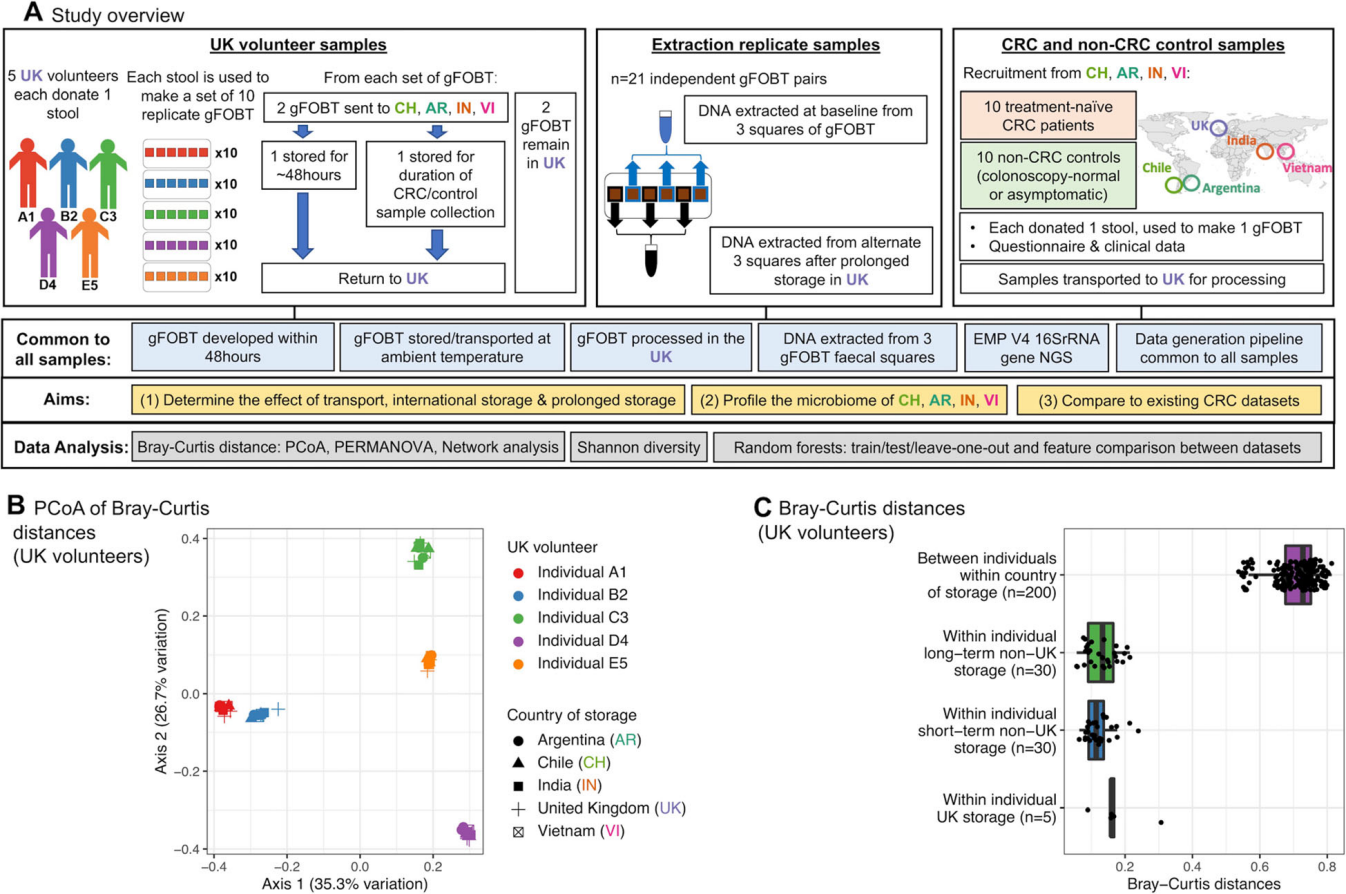
Study overview. International transport and storage has no appreciable effect on the microbiome profile of UK volunteer samples. **a** Study overview (AR = Argentina; CH = Chile; IN = India; VI = Vietnam; EMP = Earth Microbiome Project; PCoA = principle coordinate analysis). **b** PCoA of Bray-Curtis distances between UK volunteer samples. As expected, the vast majority of microbiome variation is driven by baseline inter-individual differences independently of storage location. **c** Distribution of Bray-Curtis distances between UK volunteer samples. From top to bottom: between individuals within each country of storage (Argentina, Chile, India, UK, Vietnam); within individual for samples stored in Argentina, Chile, India, or Vietnam for the duration of CRC/non-CRC control sample collection; within individual for samples stored in Argentina, Chile, India, or Vietnam for ~ 48 h; within individual for samples that remained in the UK. Again, differences due to storage are small relative to inter-individual differences

Replicate gFOBT samples were created to investigate the effect of international transport and storage (Fig. 1a). A convenience group of five healthy UK volunteers (relatives of a member of the UK research team) was used; each volunteer donated a stool. Volunteers were aged between 28 and 66, had no history of colonoscopy or antibiotic use within the preceding 6 months, and had no comorbidities. Each stool was used to make ten gFOBT (Hema Screen, Immunostics, Inc). gFOBT were stored at room temperature for 24 h. Developer solution (Hema Screen, Immunostics, Inc) was then applied and gFOBT were left to dry. gFOBT were stored in individual sealed bags at room temperature prior to transit (115–171 days).

Two gFOBT from each volunteer remained within the UK; these acted as baseline samples. Two gFOBT from each volunteer were transported at ambient temperature to India, Vietnam, Chile, and Argentina (transit time 4–8 days). Of these, one from each volunteer was stored at room temperature for a short period (2–11 days, dependent upon courier collection) before being returned to the UK (transit time 3–6 days). gFOBT were transported at ambient temperature, except for the gFOBT from Argentina which were received with ice packs due to a mistake by the courier. To limit batch effects, DNA extraction was performed upon receipt of samples from all four countries, and DNA extraction of samples which had remained in the UK was performed at the same time.

To assess the effect of the storage conditions which the CRC/non-CRC control samples had been subject to, a second set of gFOBT was stored in India, Vietnam, Chile, and Argentina at room temperature for the duration of CRC/non-CRC control sample collection (Vietnam 29 days, Argentina 76 days, India 115 days, and Chile 196 days). The recorded laboratory temperatures were as follows: UK (mean monthly temperature 20–23 °C, maximum 27 °C), Vietnam (mean not available, maximum 25 °C), Argentina (mean 15 °C, maximum 20 °C), Chile (mean 20 °C, maximum 22 °C), and India (mean 24 °C, maximum 27 °C). Samples were returned at ambient temperature with the CRC/non-CRC control samples (transit time 5–10 days), and DNA extraction was performed upon receipt.

### CRC and non-CRC control samples

gFOBT and clinical data were collected from ten CRC patients and ten non-CRC controls each from Vietnam (Can Tho University of Medicine and Pharmacy, Can Tho, April–June 2018), Argentina (Hospital Italiano de Buenos Aires, June–August 2018), India (Cancer Institute (WIA) Chennai, July–October 2018), and Chile (Universidad de los Andes Santiago, October 2018–February 2019, samples collected in fact from 11 CRC patients and ten non-CRC controls), to give a total cohort of 81 individuals (Fig. 1a). Non-CRC controls comprised either people with a normal bowel at colonoscopy (Vietnam and Argentina) or asymptomatic individuals working in the affiliated University (Chile and India). Both control groups (colonoscopy-normal controls or asymptomatic controls) are variously used in microbiome studies; we offered flexibility as the institutes in Chile and India had limited access to colonoscopy-normal controls (colonoscopy is less readily available in some developing countries). Colonoscopies were carried out a minimum of 2 weeks prior to sample collection (during which time the majority of taxa return to baseline). CRC patients were treatment-naïve. Study participants were aged over 18. Exclusion criteria included the following: antibiotic use within the preceding 6 months; foreign travel within the preceding 2 weeks; colonoscopy within the preceding 2 weeks; related to another study participant; colostomy; history of previous CRC/adenoma, colorectal surgery, pelvic radiation, or chemotherapy; known CRC syndrome or family history of hereditary CRC; and coexistent IBD or infectious bowel disease.

To limit batch effects, samples from CRC patients and non-CRC controls were collected as far as possible alternately and were transported and processed as a single batch. Participants provided a stool which was used to prepare a gFOBT. Developer solution was applied the same day in the majority of instances (occasionally the following day), and gFOBT were left to dry. gFOBT were stored in individual sealed bags at room temperature. Once collection was complete, samples were returned to the UK at ambient temperature.

### DNA extraction replicate samples

To assess the effect of prolonged UK storage at room temperature, DNA extraction replicates were created from a subset of the CRC/non-CRC control samples (*n* = 21 pairs), by extracting DNA from three squares of faecally loaded card at baseline (details below) and subsequently from the three remaining squares after prolonged UK storage (Fig. 1a). Time between DNA extraction of replicate pairs was as follows: Chilean samples 27 days, Indian samples 140 days, Argentinian samples 211 days, and Vietnamese samples 252 days.

### DNA extraction

Sample processing was performed at the University of Leeds. The QIAamp DNA Stool Mini Kit (Qiagen, Germany) was used until its discontinuation in August 2018, whereupon the equivalent QIAamp DNA Mini Kit (Qiagen, Germany) and Buffer ASL (Qiagen, Germany) were used.

From each gFOBT, three squares of faecally loaded card were dissected and processed as a single combined sample. The rationale of this approach is that it subsamples a larger volume of stool, ensuring adequate biomass even from thinly smeared gFOBT (the volume of stool per gFOBT can be very variable when prepared by study participants), and leaves three squares remaining for alternative analysis or DNA extraction replicates. Next, 800 μl of Buffer ASL was added and samples were incubated at 23 °C on a Thermomixer Comfort (Eppendorf UK) at 850 rpm for 1 h. Samples were briefly centrifuged and supernatant transferred to pathogen lysis tubes (S) (Qiagen, Germany). Samples were agitated (Vibrax VXR, IKA, UK) at a motor setting of 1800–2200 for 10 min followed by incubation at 95 °C on the Thermomixer at 850 rpm for 15 min. Samples were then centrifuged at 18625*g* for 1 min, and supernatant was transferred to a tube containing 173 μl of 10 M ammonium acetate. We then vortexed the samples and placed them on ice for 5 min, then centrifuged at 18625*g* for 5 min. Supernatant was transferred to a tube containing 725 μl of propan-2-ol, vortexed, and placed on ice for 30 min. Then, samples were centrifuged at 18625*g* for 10 min, supernatant was discarded, and 1 ml of 70% ethanol was added. Samples were centrifuged at 18625*g* for 5 min, supernatant discarded, and 500 μl 70% ethanol was added. Samples were centrifuged at 18625*g* for 3 min, supernatant discarded, and the samples left for 10 min to evaporate residual ethanol. Two hundred microliters of tris-EDTA was added, and after 10 min, samples were vortexed and added to tubes containing 200 μl of Buffer AL (QIAamp DNA Mini Kit). Fifteen microliters of Proteinase K (QIAamp DNA Mini Kit) was added; the samples were vortexed and incubated at 70 °C on the Thermomixer at 650 rpm for 10 min. The QIAamp DNA Mini Kit protocol was then followed. To elute DNA, 100 μl of UV-irradiated molecular biology grade water was added to samples for 5 min before centrifuging at 18625*g* for 1 min.

### 16S rRNA library preparation and NGS

The Earth Microbiome Project (EMP) 16S Illumina Amplicon library preparation methodology was followed [25], with Illumina 16S V4 primer constructs 515F (Parada)-806R (Apprill) [26, 27]. Single (rather than triplicate) PCR reactions were performed per sample, each with a starting amount of 20 ng DNA. One hundred fifty-two samples were sequenced as part of a total pool of 996 samples from other projects, on one lane of an Illumina HiSeq 3000, for 2x150bp sequencing, with a 10 bp index read.

### Bioinformatic processing and statistical analysis

Reads were stripped of adaptors using cutadapt [28]. Further processing was carried out in QIIME2 (version 2019.4) [29]. Reads were trimmed to a maximum of 145 bp, pairs merged, denoised, and representative sequences chosen using DADA2 [30].

Taxa were assigned to representative sequences by the QIIME2 feature classifier using the BLAST+ algorithm [31, 32], aligning sequences against the SILVA version 132 99% similarity database [33].

Within the QIIME2 environment, samples were rarified to the depth of the sample with fewest QC-passed sequences (51,000), and Shannon index alpha diversity was calculated [34], with significance assessed by the Kruskal-Wallis test [35]. Rarified samples were used to calculate Bray-Curtis beta diversity [36], and principle coordinate analysis (PCoA) was performed. Taxa, representative sequences, and distance matrices were exported from QIIME2 for analysis and graphical representation using R (version 3.5.1).

Significance of differences in beta diversity between groups was assessed by PERMANOVA analysis of Bray-Curtis distances performed using the adonis function within vegan [37]. Where necessary, multivariate models were built using repeated measures aware permutations within the PERMANOVA test, to account for repeated measures per individual (https://bitbucket.org/biobakery/hmp2_analysis/src/default/overview/src/omnibus_tests.r) [38]. Network analysis of genus level Bray-Curtis distances was performed using phyloseq [39].

To investigate the discriminatory performance of the microbiome, a random forest (RF) model [40] was built using the combined cohort, using the packages randomForest [41] and pROC [42]. To adjust for the effects of age, after per-sample normalisation, a linear model of each taxon with age was calculated, and the residual values rather than taxon abundance used to construct a RF model.

Taxa from the combined cohort were compared to other CRC faecal metagenomic datasets [23, 43–45], processed using MetaPhlAn version 3.0 [46]. The datasets contain samples from the following countries: Feng – Austria (*n* = 107); Gupta – India (*n* = 60); Thomas_a and Thomas_b – Italy (*n* = 106); Vogtmann – USA (*n* = 104); Wirbel – Germany (*n* = 125); Yachida – Japan (*n* = 518); Yu – Hong Kong, China (*n* = 128); and Zeller – France (*n* = 114). All datasets were collapsed to genus level for comparison with 16S rRNA gene amplicon data. The Thomas_c [44] dataset was merged with the Yachida [45] dataset, as both originated from the same cohort. A random forest (RF) model [40] was built from each dataset using the randomForest [41] and compared with every other dataset using pROC [42]. Area under the curve (AUC) for each validation was recorded, as were taxa importance ranks for each model. For self-vs-self comparisons, each study was randomly split into equal sized training and validation sets 20 times, and mean AUC recorded. Additionally, a leave-one-dataset-out (LODO) comparison was performed, whereby models were built from all but one dataset, and validated on the missing dataset. Finally, taxa differing significantly between groups were obtained using LEfSe (Linear discriminant analysis Effect Size) [47].

Mann-Whitney and Fisher’s exact test were performed to assess intra-country demographic differences; hypotheses were two-tailed with a significance level of 0.05. Kruskal-Wallis ANOVA was performed to assess inter-country differences of tumour size, and post-hoc Dunn *p* values with Benjamini-Hochberg FDR adjustment were calculated.

The dataset supporting the conclusions of this article is available in the ENA repository [48]: https://www.ebi.ac.uk/ena/data/view/PRJEB36789

## Results

### CRC and non-CRC control populations and gFOBT-based microbiome profiling strategies

In total, we profiled 16S rRNA gene amplicons from stool representing the gut microbiomes of 41 CRC participants and 40 non-CRC controls, spanning Argentina, Chile, India, and Vietnam (Fig. 1, Table 1). The majority of tumours were located in the caecum/ascending colon or sigmoid/rectum and were stage pT3 or pT4 (Table 2).

**Table 1:** CRC and non-CRC control characteristics

	Argentina		Chile		India		Vietnam	
	CRC	NC	CRC	NC	CRC	NC	CRC	NC
**Number of participants**	10	10	11	10	10	10	10	10
**Male**	4	6	4	4	7	6	5	3
**Median age (range)**	81 (61–89)	55.5 (37–72)	70 (56–86)	34 (22–75)	56.5 (33–73)	34 (26–45)	58 (49–88)	58.5 (37–71)
	**MW** ***p*** **= 4.4 × 10**^**−4**^		**MW** ***p*** **= 3.5 × 10**^**−3**^		**MW** ***p*** **= 1 × 10**^**−3**^			
**History of colonoscopy**^**a**^	10	10	**11**	**0**	**8**	**0**	10	10
			**FE** ***p*** **< 1 × 10**^**−5**^		**FE** ***p*** **= 7 × 10**^**−4**^			
**Medication use**	9	6	**9**	**3**	6	1	10	10
			**FE** ***p*** **= 3 × 10**^**−2**^					
**Comorbidities**^**b**^	8	4	9	5	**7**	**0**	3	3
					**FE** ***p*** **= 3.1 × 10**^**−3**^			
**Current smoker**^**c**^	0	1	0	3	1	0	3	1
**Drinks alcohol**	3	4	**4**	**9**	1	3	3	2
			**FE** ***p*** **= 2.4 × 10**^**−2**^					
**Vegetarian**	0	0	0	0	2	6	0	1

*CRC* CRC patient, *NC* non-CRC control

Mann-Whitney (MW) and Fisher’s exact test (FE) were performed to assess intra-country differences; hypotheses were two-tailed with a significance level of 0.05. Significant differences are in bold and the *p* value stated

^a^History of colonoscopy indicates whether participants had ever had a colonoscopy with bowel preparation prior to sample collection

^b^Comorbidities included in our population profile, but not as substantial analysis covariates: hypertension, gastric ulcer, gastro-oesophageal reflux disease, insulin resistance/diabetes, thyroid disease, obesity, and hypercholesterolaemia

^c^Current smoker includes participants who stopped smoking within the preceding month

**Table 2:** Tumour characteristics

	Argentina *n* = 10	Chile *n* = 11	India *n* = 10	Vietnam *n* = 10
**Tumour location**^**a**^				
**Caecum or ascending colon**	5	5	2	0
**Transverse colon**	0	0	0	1
**Descending colon**	0	0	0	1
**Sigmoid colon or rectum**	4	6	8	8
**Data not available**	1	0	0	0
**Maximum tumour size in one direction**				
**Median (cm) (range)**	**4 (0.7–7)**	**6 (2.5–11)**	**5 (3–5.5)**	**4 (3–5)**
**Data not available**	**2**	**0**	**7**	**0**
**Kruskal Wallis ANOVA** ***p*** **= 2.9 × 10**^**−2**^ **Post-hoc Dunn significant pairwise differences (Benjamini-Hochberg FDR adjusted): Chile vs Vietnam** ***p*** **= 3.5 × 10**^**−2**^				
**Tumour stage (TNM8)**^**a**^				
**T1**	0	2	0	0
**T2**	1	1	4	0
**T3**	8	8	4	0
**T4**	0	0	0	10
**Data not available**	1	0	2	0

^a^Tumour location and stage have not been tested for heterogeneity due to small numbers

Age is, of course, a substantial contributor to CRC development, although it is not generally a major driver of microbiome variation within the range studied here (e.g. associated with ~ 5% of taxonomic variation, see below). The median age of non-CRC controls from Argentina, Chile, and India was substantially younger than the corresponding CRC patients, and the median age of CRC patients from India and Vietnam was younger than that of CRC patients from Chile and Argentina (Table 1). While this did not substantially affect subsequent analyses when tested, we note it both here and in our initial profiles of microbiome composition below. Non-CRC controls from Chile and India were asymptomatic individuals working in the affiliated Universities. Non-CRC controls from Vietnam and Argentina underwent colonoscopy, yielding descriptions of ‘macroscopically normal bowel’ (*n* = 14), diverticulosis (*n* = 4), or ‘macroscopically normal bowel with haemorrhoids’ (*n* = 2), which are grouped together in subsequent analyses as non-CRC control (it should be noted that these are common colonoscopy findings in older populations and have not been associated with a distinct microbiome profile) [49] . The total reads/sample (CRC patients, non-CRC controls and UK volunteers) were 51,000–167,000 (median 117,000).

### International transport and storage of gFOBT, and prolonged storage in the UK, has no appreciable effect on results

To first investigate the effect of international transport and storage, 50 replicate gFOBT were created using stool from a subset of the total population, comprising five UK volunteers (ten replicate gFOBT/volunteer). Two gFOBT from each volunteer remained in the UK. Two gFOBT from each volunteer were transported to institutes in Argentina, Chile, India, and Vietnam; of these, one from each volunteer was stored for a short duration, and one was stored for the duration of CRC/non-CRC control sample collection (Fig. 1a).

**Fig. 2:**
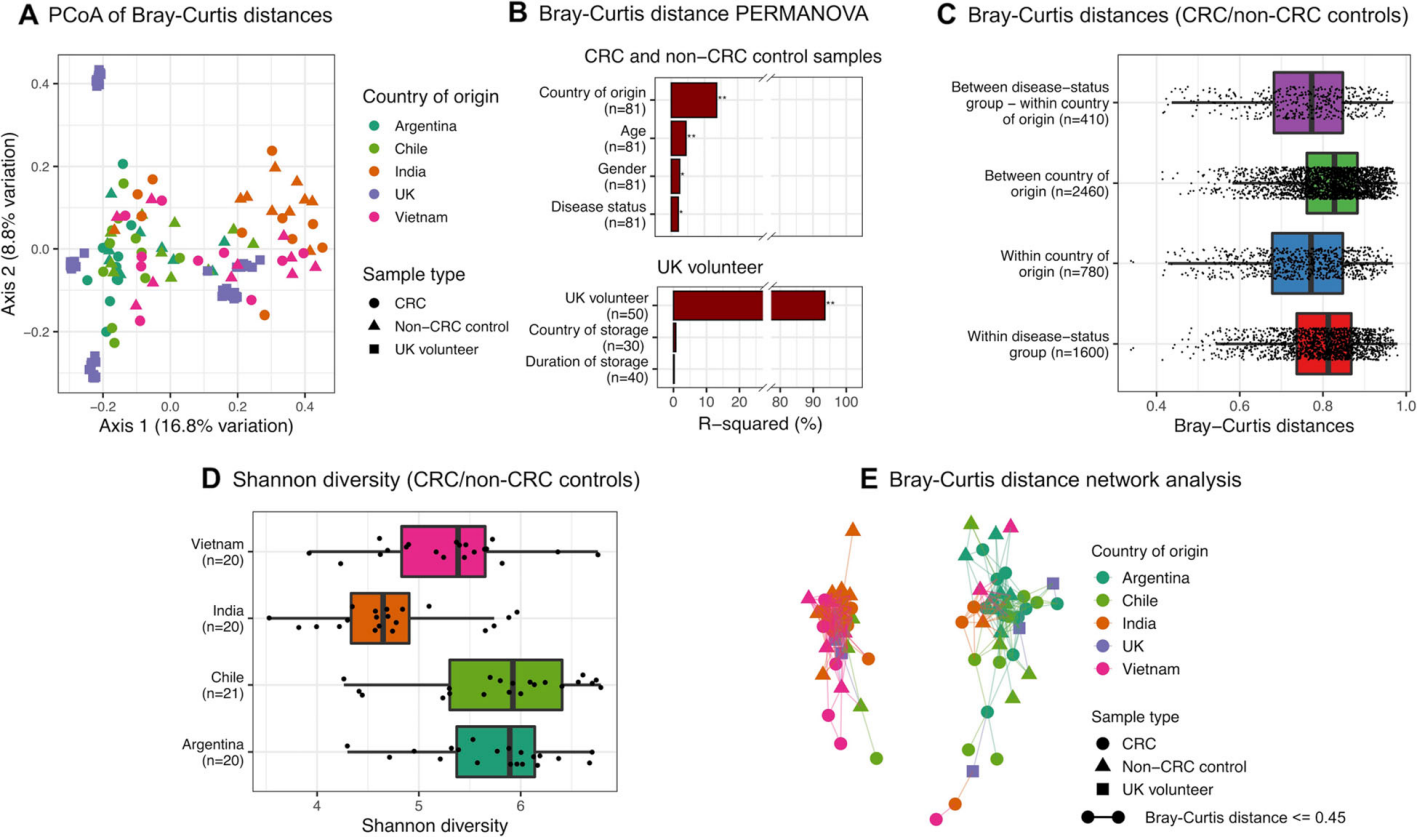
Country and continent drive microbiome structure. **a** PCoA of Bray-Curtis distances between all samples. **b** PERMANOVA based on Bray-Curtis distances between CRC and non-CRC control samples and UK volunteer samples. For the CRC/non-CRC control samples: ‘country of origin’ = Argentina, Chile, India or Vietnam; ‘disease status’ = CRC or non-CRC control. For the UK volunteer samples: ‘country of storage’ = Argentina, Chile, India, Vietnam or UK (only samples stored in the UK or short-term outside the UK were used in this analysis, as these samples underwent DNA extraction at the same time); ‘duration of storage’ = short-term or long-term storage outside the UK (samples which remained in the UK were excluded from this analysis). **p ≤ 0.01. *p ≤ 0.05. **c** Distribution of Bray-Curtis distances for CRC and non-CRC control samples: ‘disease-status group’ = CRC or non-CRC control. **d** Shannon diversity indices for CRC and non-CRC control samples. At the taxonomic resolution achievable from the data, Asian individuals generally had lower diversity than South American individuals. **e** Network representation of all samples based on Bray-Curtis distances. Each UK volunteer is represented by a single point, reflecting the average relative abundance across all samples derived from that individual. Overall dissimilarities are driven by a weak segregation between Asian and South American microbiomes

Neither country nor duration of storage had a significant effect on the microbiome structure of the UK volunteer samples, which as expected grouped by UK volunteer (Fig. 1b, c, Additional file 1: Fig. S1A). PERMANOVA of Bray-Curtis dissimilarity confirmed this, quantifying the effect of UK volunteer as *R*^2^ = 94% (*p* = 0.001) (Fig. 2b, Additional file 1: Table S1). Each UK volunteer’s taxonomic composition was assessed essentially equivalently across the different storage methods, and whilst there was a minor amount of taxonomic variability (average inter-individual replicate Bray-Curtis dissimilarity 0.13), this affected both samples which remained in the UK and samples which were transported and stored internationally (Additional file 1: Fig. S1B).

To determine whether the microbiome would remain stable if samples were stored for a prolonged period at room temperature in the UK pending DNA extraction, extraction replicates were created from a subset of the CRC/non-CRC control samples (Fig. 1a). DNA extraction was performed upon sample receipt, by dissecting three squares of faecally loaded card, and extraction replicates were created by dissecting the alternate three squares after a period of storage at room temperature. Pairs of replicate samples had similar microbiome structures and taxonomic profiles (average inter-individual replicate Bray-Curtis dissimilarity = 0.17) (Additional file 1: Fig. S2A-C). No significant taxonomic differences were detected by LEfSe between the groups of baseline and replicate samples.

### Geography drives CRC-independent gut microbiome structure of participants

Aside from inter-individual differences, the greatest determinant of microbiome structure was country of origin (Fig. 2a). PERMANOVA quantified this based on Bray-Curtis distances (*R*^2^ = 14%) (*p* = 0.001), with ‘disease status’ (CRC or non-CRC control) contributing far less (*R*^2^ = 2%) (*p* = 0.019) (Fig. 2b, c, Additional file 1: Table S1). A significant difference in alpha diversity (Shannon) was likewise detected between countries (Kruskal Wallis *p* = 4 × 10^−5^). Specifically, the alpha diversities of Vietnamese and Indian samples were significantly lower than those of the Argentinian and Chilean samples, and those of the Indian samples were lower than the Vietnamese (Fig. 2d). No significant difference in Shannon diversity index was detected between overall CRC and non-CRC control samples (Kruskal Wallis *p* = 0.28).

In addition to country of origin, continent was itself a driver of microbiome structure, with the majority of the Asian samples (India and Vietnam) distinct from the majority of the South American samples (Chile and Argentina) (Fig. 2e). In our study, similar to previous reports, Asian samples had a significantly higher relative abundance of *Prevotella* (LDA score 5.173, *p* = 2.79 × 10^−6^) and lower relative abundance of *Bacteroides* (LDA score 4.841, *p* = 2.32 × 10^−6^) compared with South American samples (Additional file 1: Fig. S3A-C).

### The CRC-associated microbiome of developing countries resembles that of developed countries

To determine the potential of the microbiome to discriminate between CRC and non-CRC control samples, a RF model was built using the combined total dataset of CRC and non-CRC controls (AUC 0.77 (CI 0.67–0.87)). Given the age-imbalance within our dataset, we compared the result with an age-adjusted RF model; the age-adjusted RF model performed equivalently (AUC 0.80 (CI 0.69–0.89)), confirming that age does not account for the discriminatory performance of the RF model.

**Fig. 3:**
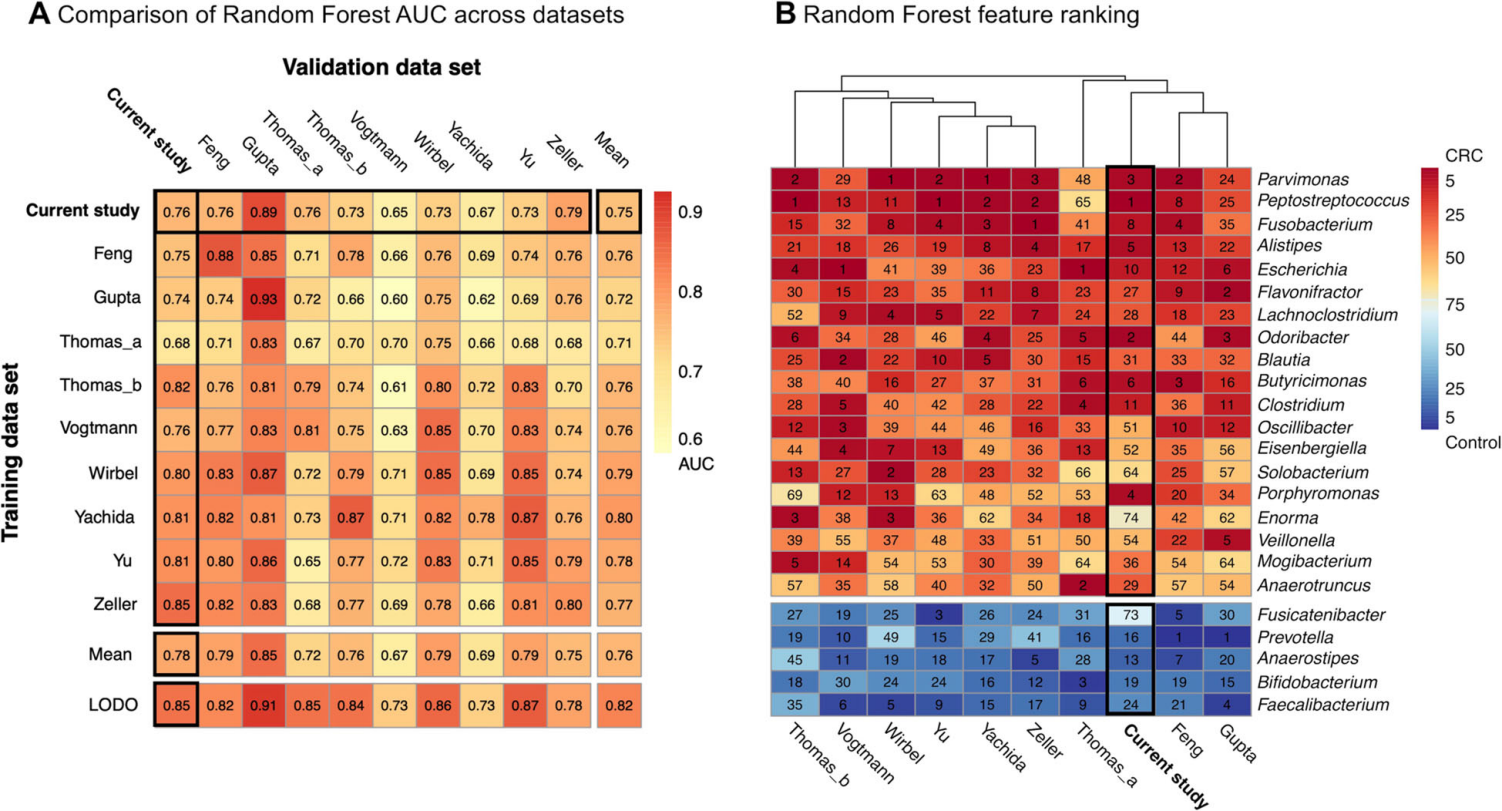
CRC prediction performance is comparable and taxonomically similar across datasets. **a** Cross-prediction matrix demonstrating random forest AUC based on genus-level relative abundances. LODO (leave-one-dataset-out) refers to the AUC generated by training a model using all but the dataset of the associated column and testing it using the dataset of that column. The datasets contain samples from the following countries: Feng – Austria; Gupta – India; Thomas_a – Italy; Thomas_b – Italy; Vogtmann – USA; Wirbel – Germany; Yachida – Japan; Yu – Hong Kong, China; Zeller – France. **b** The importance of each genus for the cross-validation performance in each dataset using gini values. Only genera in the top five in at least one dataset are shown

Next, the combined total dataset of CRC and non-CRC control amplicon profiles was compared to CRC faecal shotgun metagenomic datasets from the existing literature (Fig. 3) [23, 43–45] . These nine existing studies included 1262 total samples from the following countries: France (*n* = 114), Austria (*n* = 107), Italy (*n* = 106), Germany (*n* = 125), USA (*n* = 104), India (*n* = 60), Hong Kong, and China (*n* = 128) and Japan (*n* = 518). A random forest (RF) model trained using the current study’s amplicon dataset and tested using the external metagenomic datasets, paralleling previous methodology [44], performed strikingly similarly (mean AUC 0.75) to models trained using the external metagenomes (mean AUC 0.71–0.80) (Fig. 3a). Similarly, models trained using the external datasets performed similarly when tested using the current study’s dataset (mean AUC 0.78, leave-one-dataset-out AUC 0.85) or the other external datasets (mean AUC 0.67–0.85, leave-one-dataset-out AUC 0.73–0.91).

RF models built using each dataset ranked CRC-associated taxa of greatest discriminatory importance similarly (Fig. 3b, Additional file 1: Fig. S4A). The five CRC-associated taxa which were of greatest importance to the majority of the models (*Parvimonas*, *Peptostreptococcus*, *Fusobacterium*, *Alistipes*, and *Escherichia*) were ranked in the top ten taxa by the model built using the current study’s dataset. The five most important taxa for the current study’s model were *Peptostreptococcus*, *Odoribacter*, *Parvimonas*, *Porphyromonas*, and *Alistipes*. As mentioned, *Peptostreptococcus*, *Parvimonas*, and *Alistipes* were ranked highly by the majority of external models. In contrast, *Odoribacter* and *Porphyromonas* were ranked highly by some but not all of the external models; this discrepancy could be secondary to demographic or technical differences between cohorts. Within the current study, CRC-associated taxa differed by country; *Peptostreptococcus* and *Parvimonas* were CRC-enriched in cohorts from three of the four countries (India, Chile and Vietnam) (Additional file 1: Fig. S4B-F).

These results together indicate that the CRC-associated microbiome is substantially consistent, and can be consistently measured, across multiple very different international populations. This was true across cohorts and sample handling within this study and surprisingly also proved to hold when comparing these diverse populations to external CRC cohorts. In general, control-associated taxa were of lesser importance to the RF models than CRC-associated taxa. This is likely due to the heterogeneous nature of the current study’s control group and of different “healthy” populations’ gut microbiomes generally. It suggests that the commonality of some CRC-associated microbiome shifts may be particularly important, either causally or in response to cancer-induced changes in the intestinal microenvironment.

## Discussion

To date, limited microbiome research has been conducted in developing countries, leaving a gap in our knowledge of gut microbial relationships with chronic disease in global populations. We sought to address this in CRC by establishing a network of researchers and participants from Argentina, Chile, India, and Vietnam. First, we demonstrated that gFOBT, stored at room temperature and, importantly, also transported at ambient temperature, were suitable for genus-level faecal microbial taxonomic profiling in a translational setting across these multinational cohorts. This also remained true for gFOBT samples with prolonged storage periods at room temperature in the UK. This allowed us to further profile the faecal microbiomes of CRC patients and non-CRC controls from Argentina, Chile, India, and Vietnam, and, through comparison with external datasets, to demonstrate that the CRC-associated microbiome of these developing countries resembles that of developed countries.

As in previous methodologically similar studies in European or North American populations, the microbiome profile of replicate gFOBT samples from five UK volunteers that were either stored in the UK, or transported and stored abroad (maximum temperature 27 °C) for either a short or long (maximum 196 days) duration, was predominantly shaped by inter-individual differences and not appreciably by storage conditions. Similarly, DNA extraction replicate pairs demonstrated consistent microbiome profiles after UK room temperature storage (maximum 252 days). Together, these results indicate that sample analysis did not suffer due to bacterial overgrowth or DNA degradation, despite prolonged storage and transportation at ambient temperature. This is presumably due to the action of Hema Screen developer solution (which contains a stabilised mixture of hydrogen peroxide (< 6%) and 75% denatured ethyl alcohol in aqueous solution); notably, developer would not normally be added to screening gFOBT samples immediately upon collection, although a rapid fixation process is easily applicable in field settings. Although not formally assessed by this study, it is expected that an equivalent alcohol/hydrogen-peroxide based solution could be readily made in-house at low-cost, facilitating microbiome-research in developing countries.

Our results agree with and build on existing studies which have also demonstrated the suitability of gFOBT for microbial community amplicon profiling, aiming to validate such methods not only for research but for field clinical settings [9–15]. Our study adds to the small number which have assessed stability after prolonged storage, by demonstrating stability after eight months for 21 replicate pairs. Two studies investigated the microbiome of card samples at higher ambient temperatures; one showed stability of FTA at 4–40 °C [16] and the other showed stability of gFOBT stored for four days at ambient temperature in Bangladesh [17]. Our study corroborates these findings; additionally, we show that *prolonged* storage at room temperature in developing countries and *international transport* of samples at ambient temperature has no detrimental effect. The latter in particular is an important finding, offering an alternative to fresh and/or frozen samples that is practical for use at population scale in international clinical settings at reduced cost. While the types and detail of microbiome profiles that can be obtained from such samples remains limited, it is conversely appropriate for some important population-scale applications such as cancer biomarker testing. We hope these findings will thus encourage others to consider using gFOBT for appropriate epidemiology in settings where more detailed sample types are infeasible.

As expected, we found that for the CRC and non-CRC control samples, the greatest determinant of microbiome structure, aside from inter-individual variation, was country of origin, corresponding to 14% of overall microbial variation. This is in agreement with a previous study, in which country of origin accounted for a similar amount of variation (*R*^2^ = 22%) [50]. Additionally, countries within continents also showed greater microbial similarity [51, 52]. Inter-continent differences in microbiome structure were likely a consequence of the inverse *Prevotella* to *Bacteroides* ratio of Asian and South American samples. Similar results have been previously documented by microbiome studies of healthy Indians, Argentinians, and Chileans and are in keeping with expected differences between these countries [52–58]. The fact that the Asian samples had lower alpha diversities was somewhat surprising. However, other studies have also demonstrated low alpha diversity of faecal samples from healthy Indians, perhaps due to the high relative abundance of *Prevotella* [54, 56], as clades such as these and *Bacteroides* can be difficult to distinguish in amplicon-based profiling (thus leading to lower apparent diversity when dominant).

By comparing a random forest model built from our dataset with models built using external, largely developed cohorts, we demonstrated a surprising commonality of the CRC-associated microbiome, particularly CRC-associated taxa, between developed and developing populations. This finding is remarkable in light of technical differences between the studies (method of sample collection and amplicon versus metagenomic sequencing, to name the largest) and the fact that we combined CRC patients and non-CRC controls from four developing countries with distinct microbiome profiles. It should be noted, however, that whilst the direction of the effect is similar between our study and larger, more homogeneous, later-stage CRC cohorts, the magnitude of the effect is understandably smaller. The five taxa which were of greatest importance to the majority of these models were *Parvimonas*, *Peptostreptococcus*, *Fusobacterium*, *Alistipes*, and *Escherichia*. Although we were limited in the precision with which we could measure them, within these genera previous studies have associated several of their species with CRC: *Peptostreptococcus stomatis* [23], *Peptostreptococcus anaerobius* [45], *Fusobacterium nucleatum* [59], *Parvimonas micra* [59], *Alistipes finegoldii* [59], and *pks+ Escherichia coli* [2]. Of these, *Fusobacterium nucleatum* and *pks+ Escherichia coli* in particular have been suggested as putative ‘oncomicrobes’*. Fusobacterium nucleatum* has been shown to promote tumour proliferation, pro-tumour inflammation and to subvert anti-tumour immune responses [60], whilst colibactin, produced by *pks+ Escherichia coli*, has been shown to cause DNA damage [2].

Outside of these examples, many of the additional CRC-enriched taxa are oral bacteria that rarely colonise the gut during ‘health’ but have been implicated in a variety of inflammatory and dysbiotic conditions [61]. It has been hypothesised that oral microbial growth in the colon can cause increased mucosal permeability, with subsequent bacterial invasion, inflammation, and epithelial proliferation, and indeed associated biofilms have been shown to induce tumourigenesis in a mouse model [62–64]. Whether cause or consequence of tumour formation, the fact that these bacteria are found in both developed and developing cohorts points towards the oral microbiome as a shared source of CRC-associated taxa. Geographical differences of the oral microbiome have been described, but the universality of CRC-associated taxa derived from the oral microbiome has not, to our knowledge, been extensively investigated [65].

Continuing to explore the global effects of the microbiome on CRC has the potential to improve both the disease’s management worldwide and our understanding of the underlying basic biology. It will be important to expand the cohort by sampling a larger number of participants with more rigorous age and gender matching, in addition to expanding the number of countries profiled, as well as the geographical catchment within countries, many of which show great intra-country diversity. Importantly, microbiome profiling may provide valuable insight into the rising incidence of CRC within these countries, and the shared CRC-associated microbiome raises the potential of a generalisable microbiome-based CRC screening test. To this end, we have demonstrated that gFOBT is a suitable method of faecal sample collection for 16S rRNA gene research in developing countries (Argentina, Chile, India, and Vietnam) and that their CRC-associated microbiome shares many features with that of developed countries. We encourage other researchers to investigate the CRC microbiome in greater depth and in additional populations, with the goal of preventing or treating the disease around the globe.

## Conclusions

Limited CRC-microbiome research has been conducted in developing countries, yet CRC incidence is increasing in these areas. One of the impediments to creating and applying CRC-microbiome biomarkers is the collection of frozen stool samples. Here, we investigated the efficacy of stool-based biomarkers using bowel cancer screening cards (gFOBT), stored and, importantly, transported at room temperature. We then used this technique to investigate the microbiome of CRC patients and controls from four developing countries (Argentina, Chile, India, and Vietnam). Remarkably, we show that the CRC-associated microbiome of these developing countries resembles that of developed countries, even when using limited, field-appropriate, and scalable sampling methods.

## Supplementary Information

**Additional file 1: Table S1.** PERMANOVA analysis. *P*-values < 0.05 are shaded grey. R^2^ values are recorded to two decimal places. **Fig. S1A.** Distribution of Bray-Curtis distances between UK volunteer samples. The five UK volunteers are labelled A1-E5. Within individual Bray-Curtis distances are low, despite differences in sample storage. **Fig. S1B.** Genus-level taxonomic profile of UK volunteer samples. Each bar represents a sample labelled as: UK volunteer (A1-E5); country of storage (AR = Argentina; CH = Chile; IN = India; VI = Vietnam; UK); storage duration (S = short-term storage; L = long-term storage (i.e. the duration of CRC/non-CRC control sample collection); R = samples which remained in the UK). The key contains the top 20 taxa (where a genus was described as family_group, groups were merged and only the family name is included for brevity); additional taxa are coloured grey. There is minimal taxonomic variability between samples from the same individual, and taxonomic variability affects both samples which remained in the UK and samples which were transported and stored internationally. **Fig. S2A.** PCoA of Bray-Curtis distances for extraction replicates. Points are coloured as extraction replicate pairs. **Fig. S2B.** Distribution of Bray-Curtis distances between extraction replicate samples. **Fig. S2C.** Genus-level taxonomic profile of extraction replicate samples. Each bar represents a sample labelled as follows: country of origin (AR = Argentina; CH = Chile; IN = India; VI = Vietnam); disease status (CRC = CRC; NC = non-CRC control); sample ID; whether the sample is an extraction replicate (indicated by .R). For ease of comparison, taxa are coloured as per Supplementary Fig. 1B. Replicate pairs have similar taxonomic profiles. **Fig. S3A.** Genus-level taxonomic profile of CRC and non-CRC control samples. Each bar represents a sample labelled as follows: country of origin (AR = Argentina; CH = Chile; IN = India; VI = Vietnam); disease status (CRC = CRC; NC = non-CRC control). For ease of comparison, taxa are coloured as per Supplementary Fig. 1B. South American samples generally have a high relative abundance of *Bacteroides*, and Asian samples a high relative abundance of *Prevotella*. **Fig. S3B.** The mean taxonomic composition (genus-level) of CRC and non-CRC control samples. Each bar represents the mean taxonomic composition of a group labelled as follows: country of origin (AR = Argentina; CH = Chile; IN = India; VI = Vietnam); disease status (CRC = CRC; NC = non-CRC control). For ease of comparison, taxa are coloured as per Supplementary Fig. 1B. South American samples have a high relative abundance of *Bacteroides*, and Asian samples a high relative abundance of *Prevotella*. **Fig. S3C.** LEfSe plot illustrating taxa enriched in South American (SA) compared with Asian (AS) samples. **Fig. S4A.** Distributions of relative abundance of genera of greatest importance to random forest models. The boxplots labelled ‘All’ are a summary of all of the studies, including the current study. The first 19 taxa are CRC-enriched (mean relative abundance) in the majority of studies; the final 5 taxa are control-enriched (mean relative abundance) in the majority of studies. **Fig. S4B**. LEfSe plot illustrating taxa enriched in CRC compared with non-CRC controls for the current study cohort as a whole. **Fig. S4C.** LEfSe plot illustrating taxa enriched in CRC compared with non-CRC controls (Argentina). **Fig. S4D.** LEfSe plot illustrating taxa enriched in CRC compared with non-CRC controls (Chile). **Fig. S4E**. LEfSe plot illustrating taxa enriched in CRC compared with non-CRC controls (India). **Fig. S4F.** LEfSe plot illustrating taxa enriched in CRC compared with non-CRC controls (Vietnam).

## Data Availability

The dataset (16S rRNA gene V4 amplicon data from faecal samples) supporting the conclusions of this article is available in the ENA repository [48]: https://www.ebi.ac.uk/ena/data/view/PRJEB36789

## Abbreviations

AUC: Area under receiver operator characteristic curve
CRC: Colorectal cancer
DAC: Development Assistance Committee
EMP: Earth Microbiome Project
FTA: Flinders Technology Associates cards
gFOBT: Guaiac faecal occult blood test
LEfSe: Linear discriminant analysis Effect Size
LODO: Leave-one-dataset-out
PCoA: Principle coordinate analysis
RF: Random forest
