# Supplementary material for: The colorectal cancer-associated faecal microbiome of developing countries resembles that of developed countries
Source: Genome Med. 2021 Feb 16;13:27. doi: 10.1186/s13073-021-00844-8 (PMC7887780; doi:10.1186/s13073-021-00844-8)
Supplement: Supplementary file 1 — Additional file 1: Table S1. PERMANOVA analysis. P-values < 0.05 are shaded grey. R2 values are recorded to two decimal places. Fig. S1A. Distribution of Bray-Curtis distances between UK volunteer samples. The five UK volunteers are labelled A1-E5. Within individual Bray-Curtis distances are low, despite differences in sample storage. Fig. S1B. Genus-level taxonomic profile of UK volunteer samples. Each bar represents a sample labelled as: UK volunteer (A1-E5); country of storage (AR = Argentina; CH = Chile; IN = India; VI = Vietnam; UK); storage duration (S = short-term storage; L = long-term storage (i.e. the duration of CRC/non-CRC control sample collection); R = samples which remained in the UK). The key contains the top 20 taxa (where a genus was described as family_group, groups were merged and only the family name is included for brevity); additional taxa are coloured grey. There is minimal taxonomic variability between samples from the same individual, and taxonomic variability affects both samples which remained in the UK and samples which were transported and stored internationally. Fig. S2A. PCoA of Bray-Curtis distances for extraction replicates. Points are coloured as extraction replicate pairs. Fig. S2B. Distribution of Bray-Curtis distances between extraction replicate samples. Fig. S2C. Genus-level taxonomic profile of extraction replicate samples. Each bar represents a sample labelled as follows: country of origin (AR = Argentina; CH = Chile; IN = India; VI = Vietnam); disease status (CRC = CRC; NC = non-CRC control); sample ID; whether the sample is an extraction replicate (indicated by .R). For ease of comparison, taxa are coloured as per Supplementary Fig. 1B. Replicate pairs have similar taxonomic profiles. Fig. S3A. Genus-level taxonomic profile of CRC and non-CRC control samples. Each bar represents a sample labelled as follows: country of origin (AR = Argentina; CH = Chile; IN = India; VI = Vietnam); disease status (CRC = CRC; NC = non-CRC co [file 13073_2021_844_MOESM1_ESM.pdf]

Additional Tables and Figures

Table S1. PERMANOVA analysis. P-values <0.05 are shaded grey. R^2 values are recorded to two decimal places.

CRC and non-CRC control samples		
	**p-value**	**R^2**
Country of origin (n=81)	0.001	13.71
Disease status (n=81)	0.019	2.14
Age (n=81)	0.001	4.45
Gender (n=81)	0.011	2.55
**UK volunteer samples**		
	**p-value**	**R^2**
UK volunteer (n=50)	0.001	93.53
Duration of storage (n=40)	0.253	0.20
Country of storage (n=30)	0.971	0.70

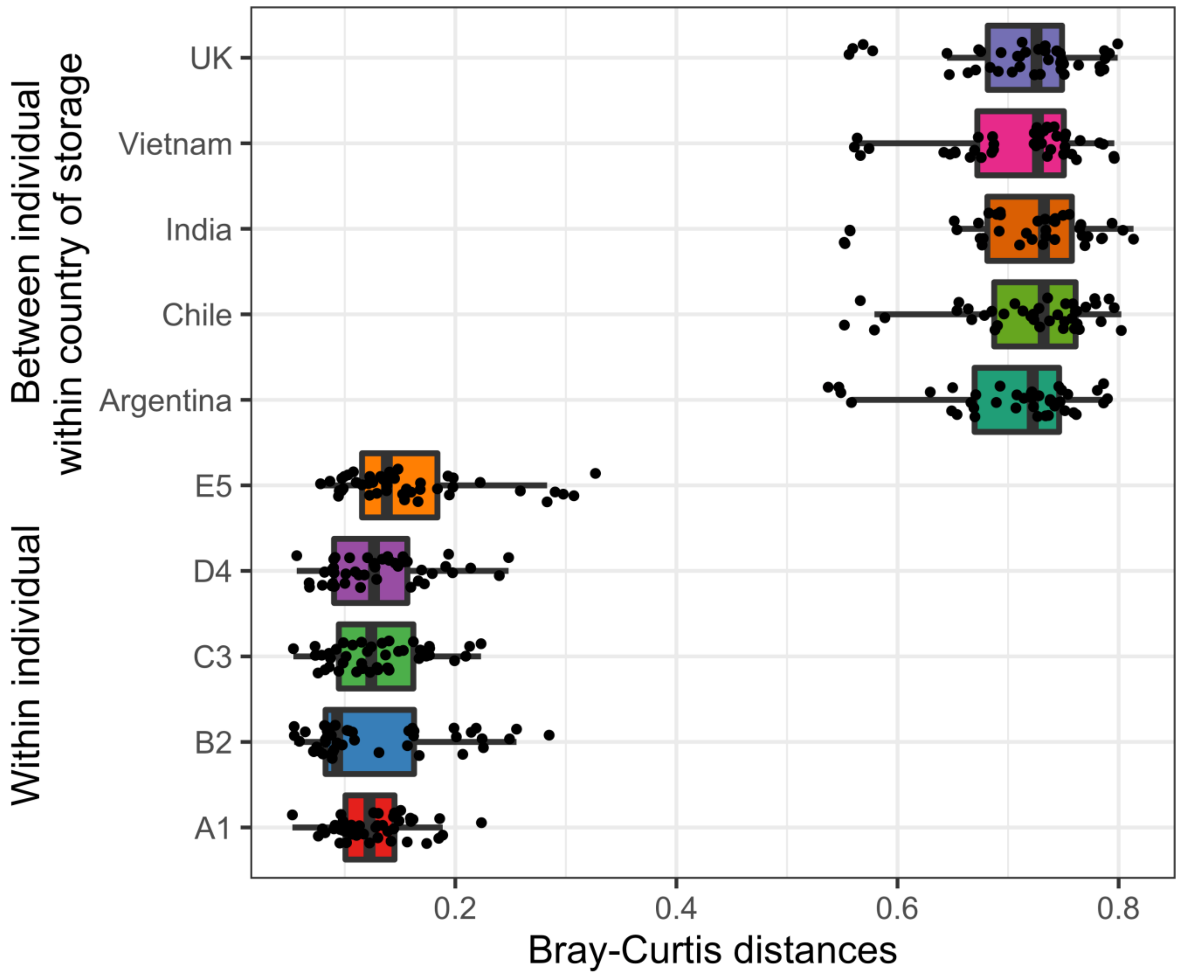

Figure S1A. Distribution of Bray-Curtis distances between UK volunteer samples. The five UK volunteers are labelled A1-E5. Within individual Bray-Curtis distances are low, despite differences in sample storage.

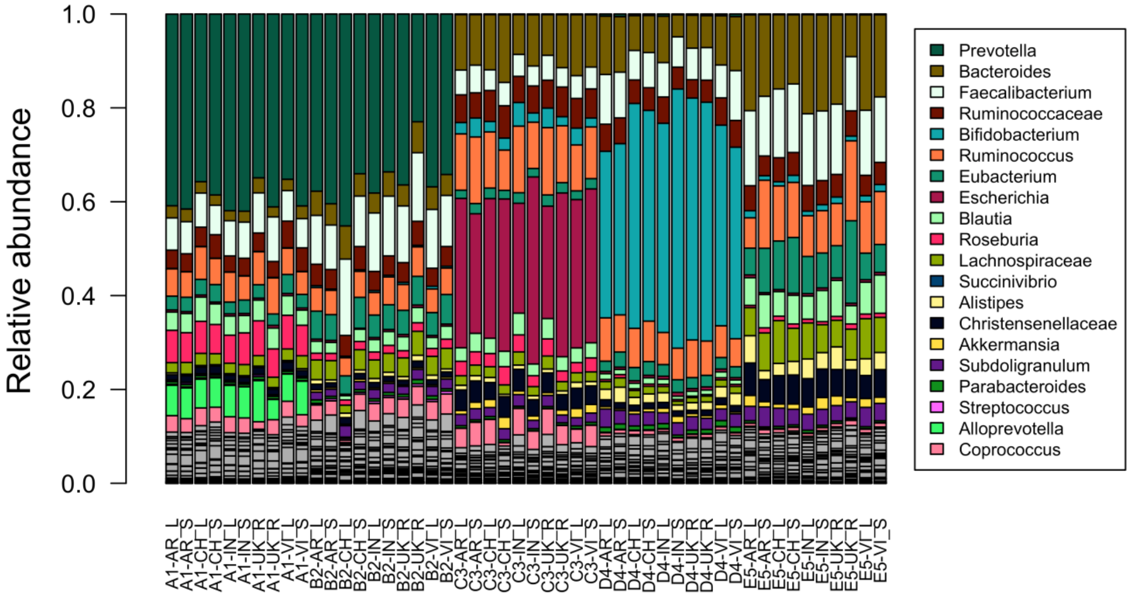

Figure S1B. Genus-level taxonomic profile of UK volunteer samples. Each bar represents a sample labelled as: UK volunteer (A1-E5); country of storage (AR = Argentina; CH = Chile; IN = India; VI = Vietnam; UK); storage duration (S = short-term storage; L = long-term storage (i.e. the duration of CRC/non-CRC control sample collection); R = samples which remained in the UK). The key contains the top 20 taxa (where a genus was described as family_group, groups were merged and only the family name is included for brevity); additional taxa are coloured grey. There is minimal taxonomic variability between samples from the same individual, and taxonomic variability affects both samples which remained in the UK and samples which were transported and stored internationally.

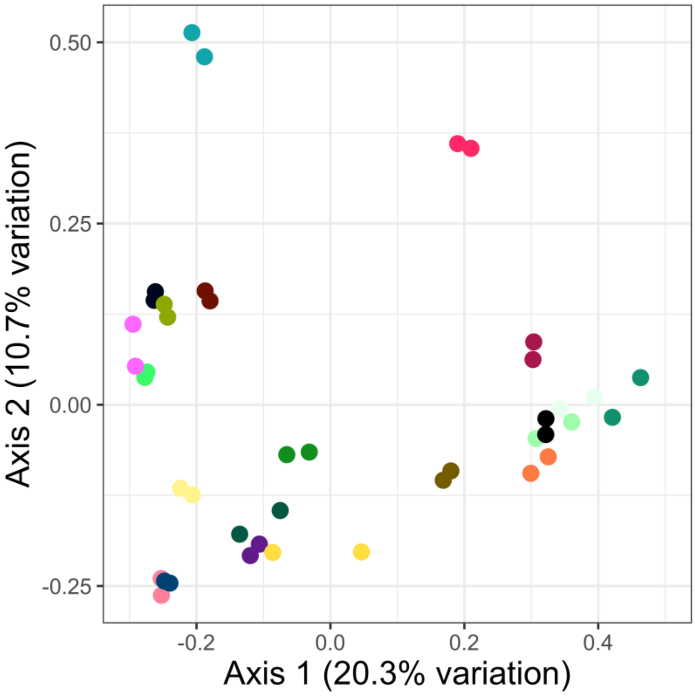

Figure S2A. PCoA of Bray-Curtis distances for extraction replicates. Points are coloured as extraction replicate pairs.

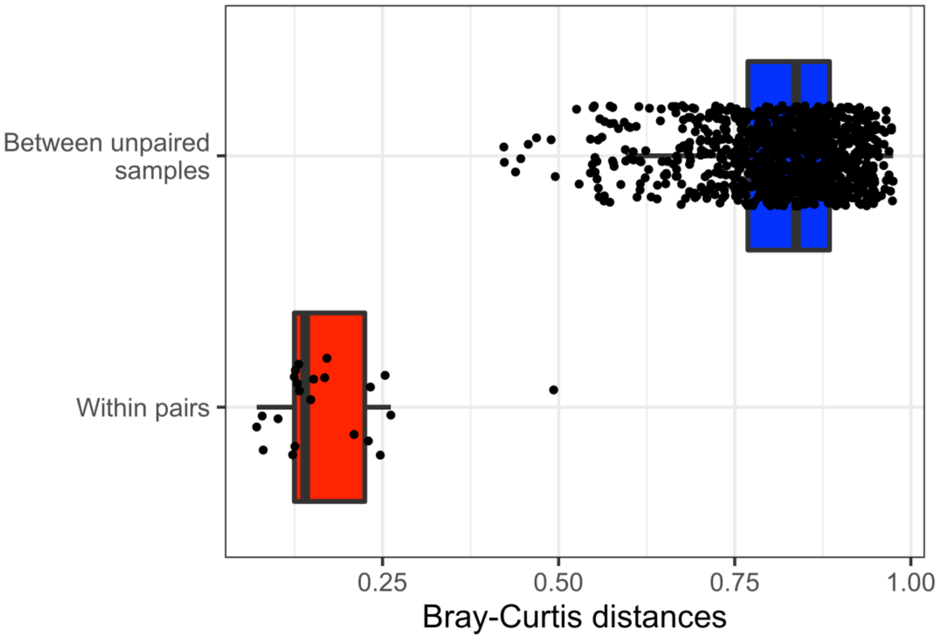

Figure S2B. Distribution of Bray-Curtis distances between extraction replicate samples.

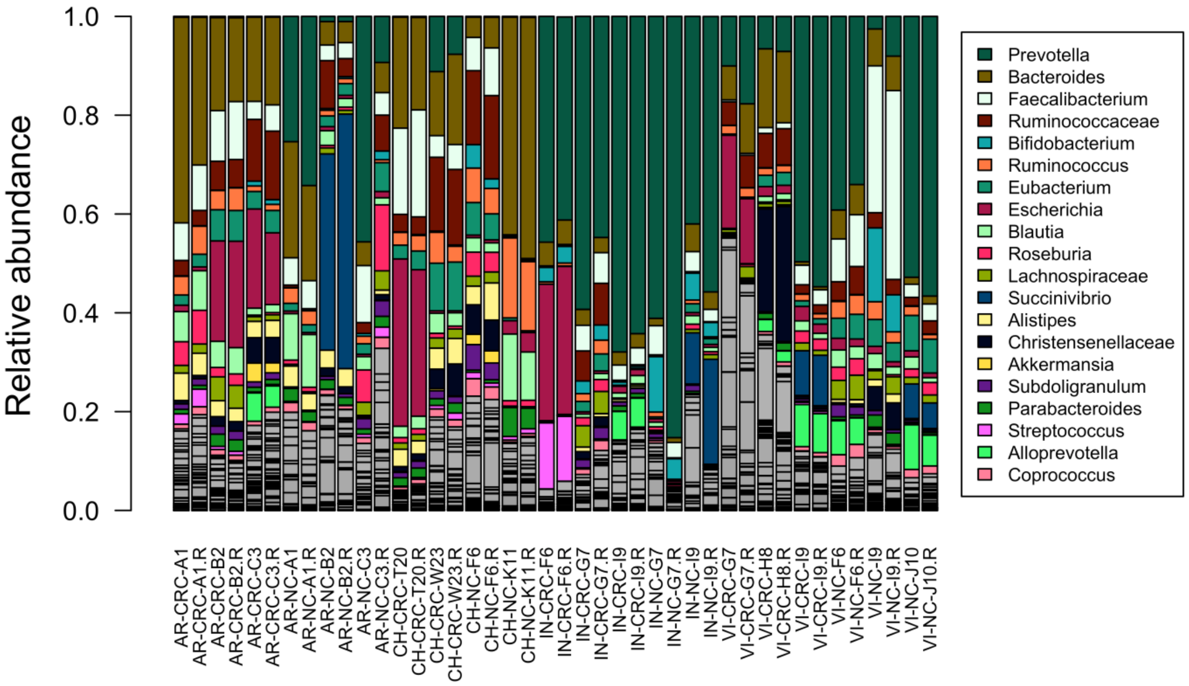

Figure S2C. Genus-level taxonomic profile of extraction replicate samples. Each bar represents a sample labelled as follows: country of origin (AR = Argentina; CH = Chile; IN = India; VI = Vietnam); disease status (CRC = CRC; NC = non-CRC control); sample ID; whether the sample is an extraction replicate (indicated by .R). For ease of comparison, taxa are coloured as per Supplementary Figure 1B. Replicate pairs have similar taxonomic profiles.

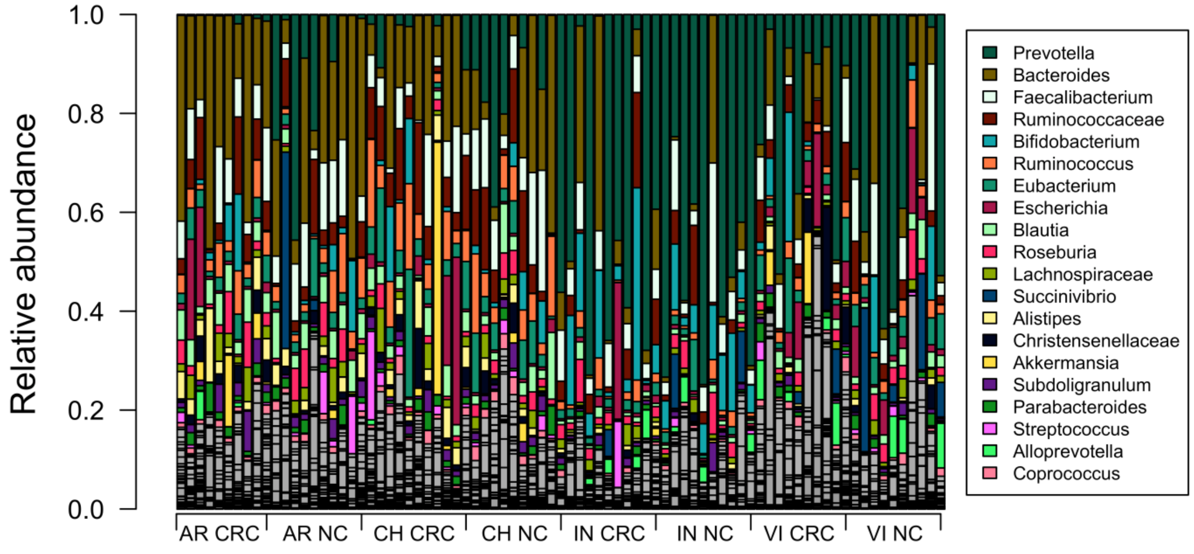

Figure S3A. Genus-level taxonomic profile of CRC and non-CRC control samples. Each bar represents a sample labelled as follows: country of origin (AR = Argentina; CH = Chile; IN = India; VI = Vietnam); disease status (CRC = CRC; NC = non-CRC control). For ease of comparison, taxa are coloured as per Supplementary Figure 1B. South American samples generally have a high relative abundance of *Bacteroides*, and Asian samples a high relative abundance of *Prevotella*.

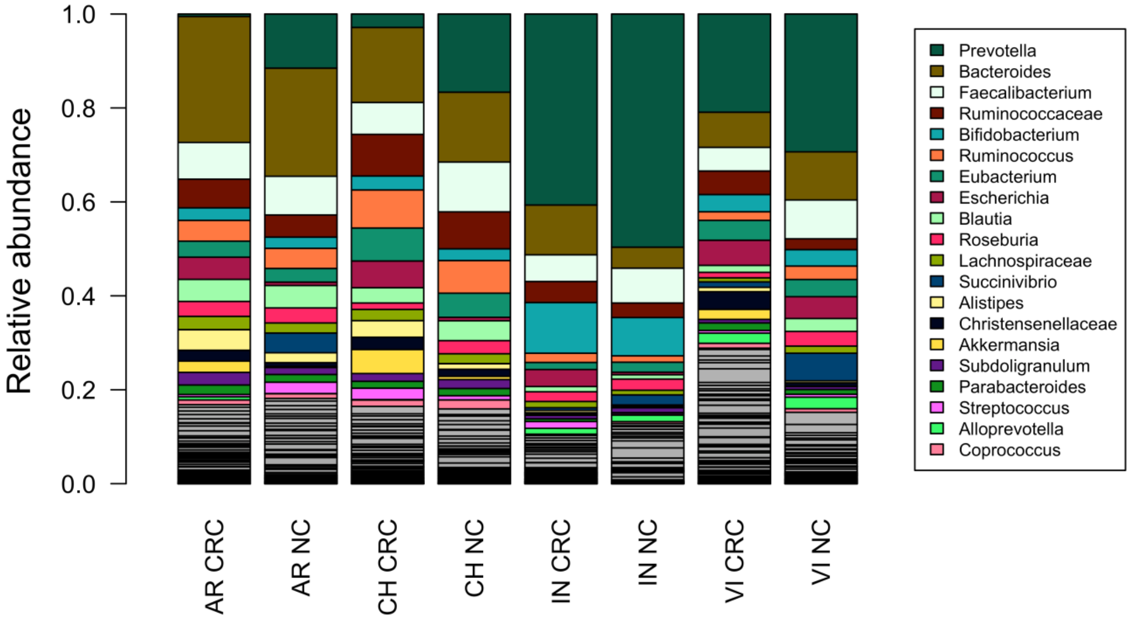

Figure S3B. The mean taxonomic composition (genus-level) of CRC and non-CRC control samples. Each bar represents the mean taxonomic composition of a group labelled as follows: country of origin (AR = Argentina; CH = Chile; IN = India; VI = Vietnam); disease status (CRC = CRC; NC = non-CRC control). For ease of comparison, taxa are coloured as per Supplementary Figure 1B. South American samples have a high relative abundance of *Bacteroides*, and Asian samples a high relative abundance of *Prevotella*.

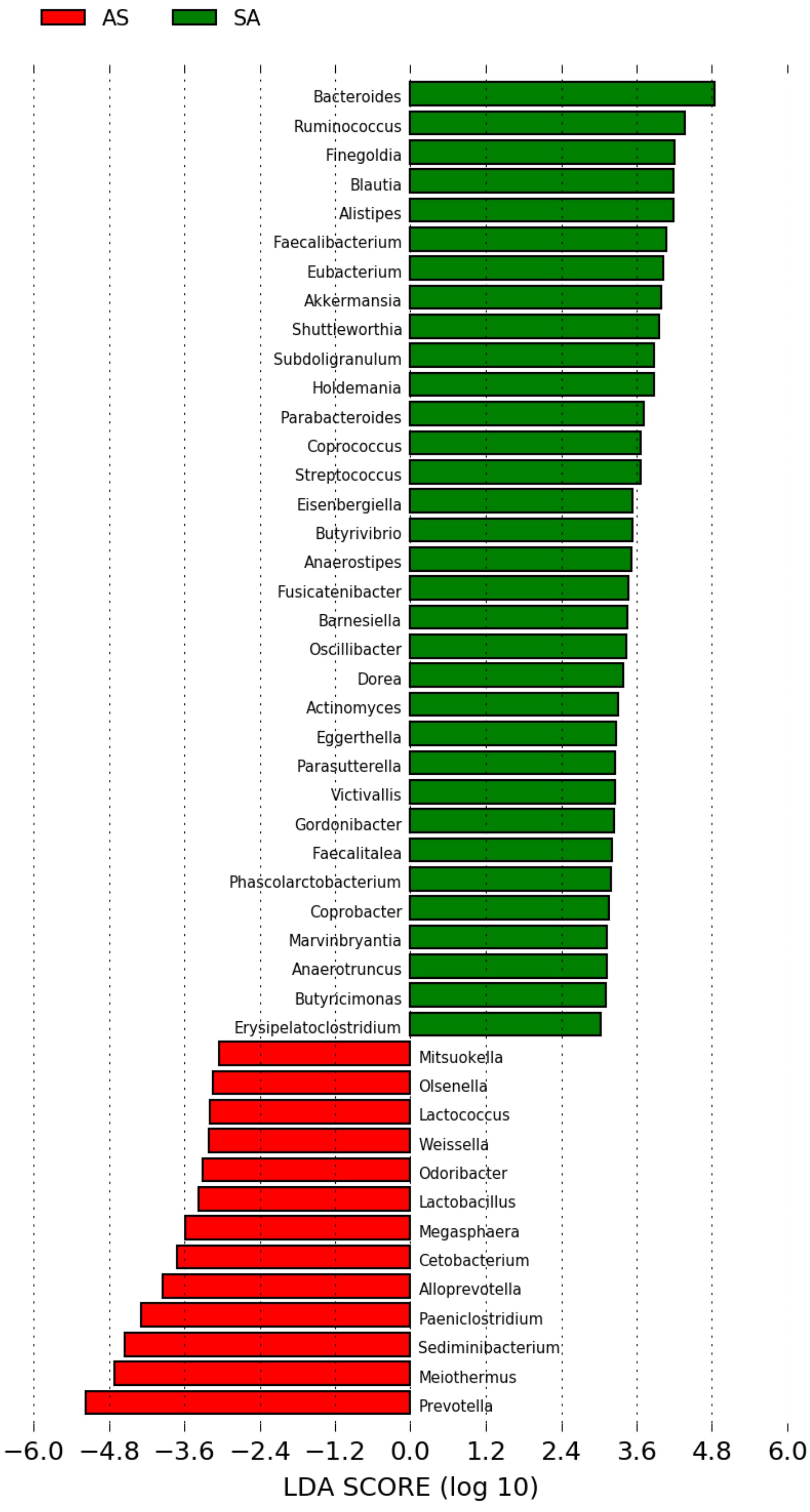

Figure S3C. LEfSe plot illustrating taxa enriched in South American (SA) compared with Asian (AS) samples.

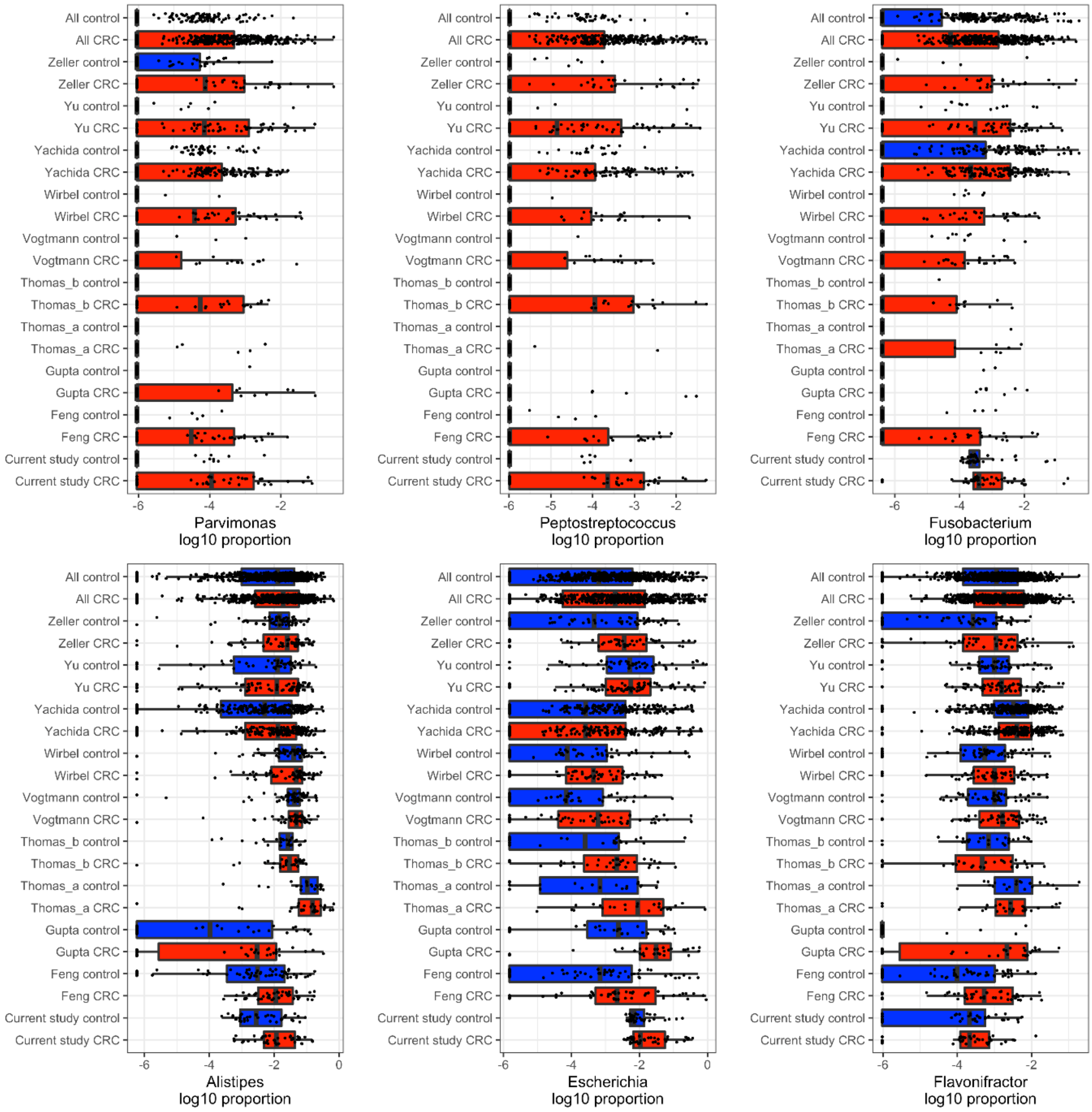

All control
All CRC
Zeller control
Zeller CRC
Yu control
Yu CRC
Yachida control
Yachida CRC
Wirbel control
Wirbel CRC
Vogtmann control
Vogtmann CRC
Thomas_b control
Thomas_b CRC
Thomas_a control
Thomas_a CRC
Gupta control
Gupta CRC
Feng control
Feng CRC
Current study control
Current study CRC
-6
-4
-2
Parvimonas
log10 proportion
All control
All CRC
Zeller control
Zeller CRC
Yu control
Yu CRC
Yachida control
Yachida CRC
Wirbel control
Wirbel CRC
Vogtmann control
Vogtmann CRC
Thomas_b control
Thomas_b CRC
Thomas_a control
Thomas_a CRC
Gupta control
Gupta CRC
Feng control
Feng CRC
Current study control
Current study CRC
-6
-5
-4
-3
-2
Peptostreptococcus
log10 proportion
All control
All CRC
Zeller control
Zeller CRC
Yu control
Yu CRC
Yachida control
Yachida CRC
Wirbel control
Wirbel CRC
Vogtmann control
Vogtmann CRC
Thomas_b control
Thomas_b CRC
Thomas_a control
Thomas_a CRC
Gupta control
Gupta CRC
Feng control
Feng CRC
Current study control
Current study CRC
-6
-4
-2
Fusobacterium
log10 proportion
All control
All CRC
Zeller control
Zeller CRC
Yu control
Yu CRC
Yachida control
Yachida CRC
Wirbel control
Wirbel CRC
Vogtmann control
Vogtmann CRC
Thomas_b control
Thomas_b CRC
Thomas_a control
Thomas_a CRC
Gupta control
Gupta CRC
Feng control
Feng CRC
Current study control
Current study CRC
-6
-4
-2
0
Alistipes
log10 proportion
All control
All CRC
Zeller control
Zeller CRC
Yu control
Yu CRC
Yachida control
Yachida CRC
Wirbel control
Wirbel CRC
Vogtmann control
Vogtmann CRC
Thomas_b control
Thomas_b CRC
Thomas_a control
Thomas_a CRC
Gupta control
Gupta CRC
Feng control
Feng CRC
Current study control
Current study CRC
-6
-4
-2
0
Escherichia
log10 proportion
All control
All CRC
Zeller control
Zeller CRC
Yu control
Yu CRC
Yachida control
Yachida CRC
Wirbel control
Wirbel CRC
Vogtmann control
Vogtmann CRC
Thomas_b control
Thomas_b CRC
Thomas_a control
Thomas_a CRC
Gupta control
Gupta CRC
Feng control
Feng CRC
Current study control
Current study CRC
-6
-4
-2
Flavonifractor
log10 proportion

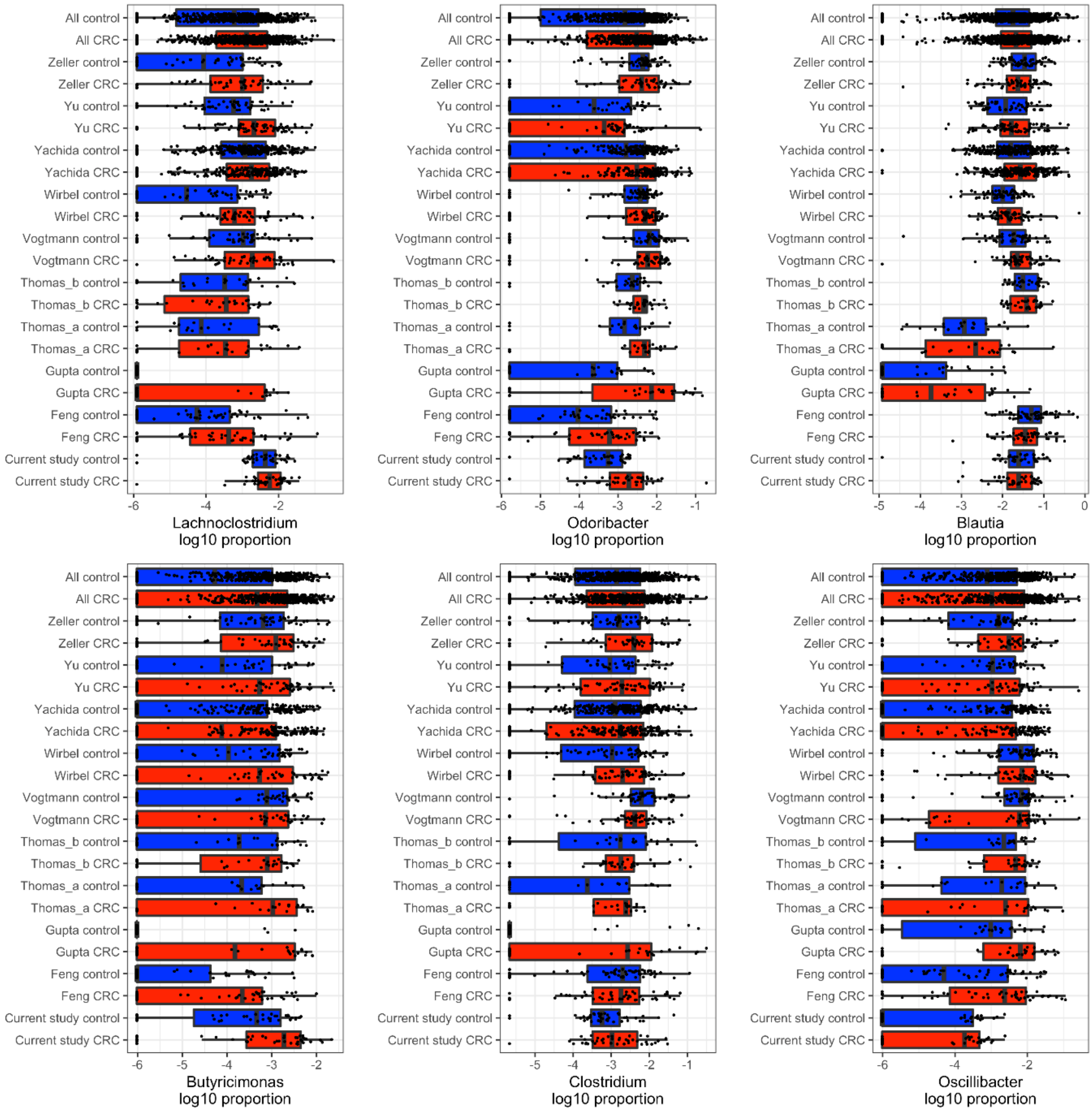

All control
All CRC
Zeller control
Zeller CRC
Yu control
Yu CRC
Yachida control
Yachida CRC
Wirbel control
Wirbel CRC
Vogtmann control
Vogtmann CRC
Thomas_b control
Thomas_b CRC
Thomas_a control
Thomas_a CRC
Gupta control
Gupta CRC
Feng control
Feng CRC
Current study control
Current study CRC
-6 -4 -2
Lachnoclostridium
log10 proportion
-6 -5 -4 -3 -2 -1
Odoribacter
log10 proportion
-5 -4 -3 -2 -1 0
Blautia
log10 proportion
-6 -5 -4 -3 -2
Butyricimonas
log10 proportion
-5 -4 -3 -2 -1
Clostridium
log10 proportion
-6 -4 -2
Oscillibacter
log10 proportion

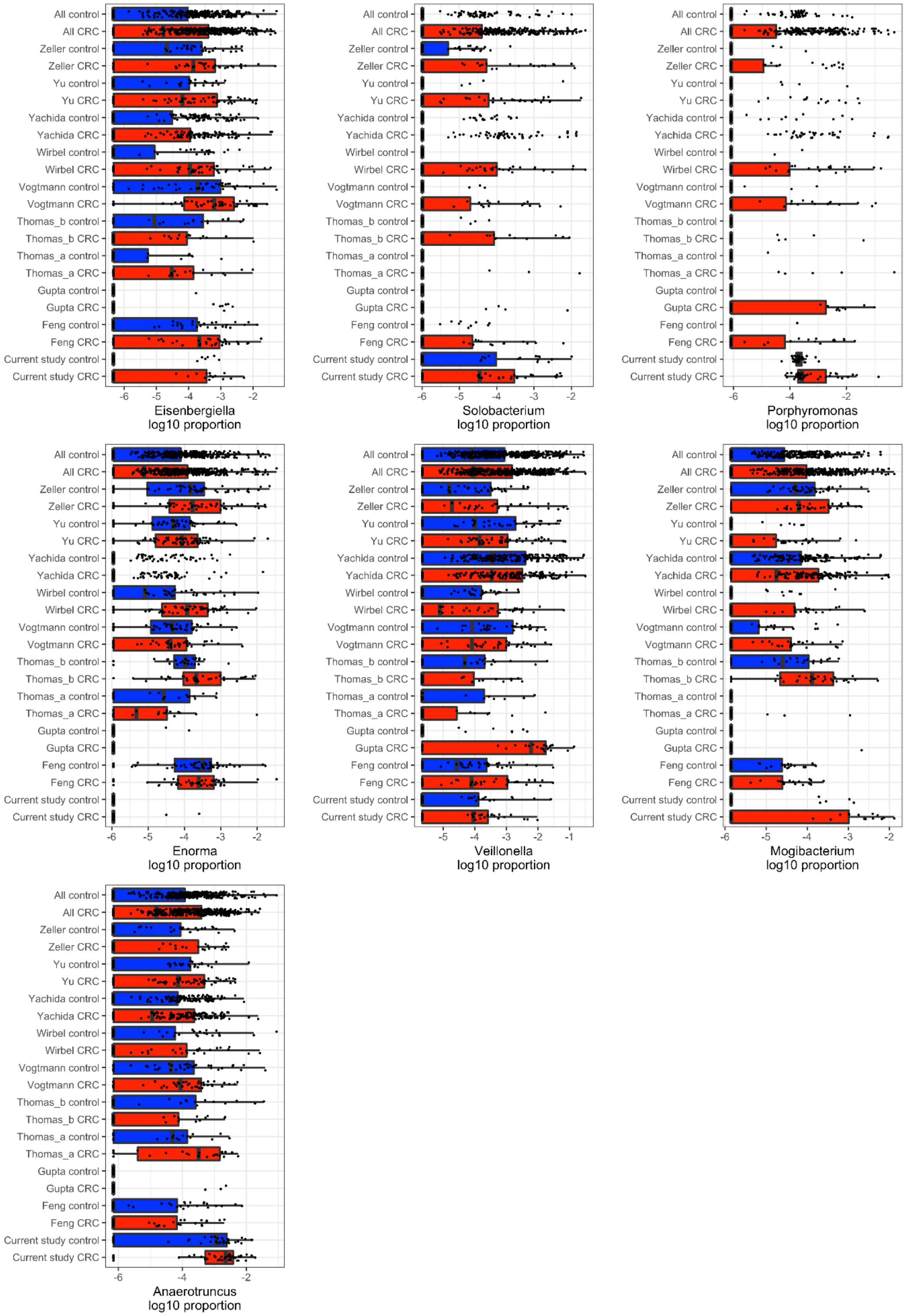

All control
All CRC
Zeller control
Zeller CRC
Yu control
Yu CRC
Yachida control
Yachida CRC
Wirbel control
Wirbel CRC
Vogtmann control
Vogtmann CRC
Thomas_b control
Thomas_b CRC
Thomas_a control
Thomas_a CRC
Gupta control
Gupta CRC
Feng control
Feng CRC
Current study control
Current study CRC
-6 -5 -4 -3 -2
Eisenbergiella
log10 proportion
-6 -5 -4 -3 -2
Solobacterium
log10 proportion
-6 -4 -2
Porphyromonas
log10 proportion
-6 -5 -4 -3 -2
Enorma
log10 proportion
-5 -4 -3 -2 -1
Veillonella
log10 proportion
-6 -5 -4 -3 -2
Mogibacterium
log10 proportion
-6 -4 -2
Anaerotruncus
log10 proportion

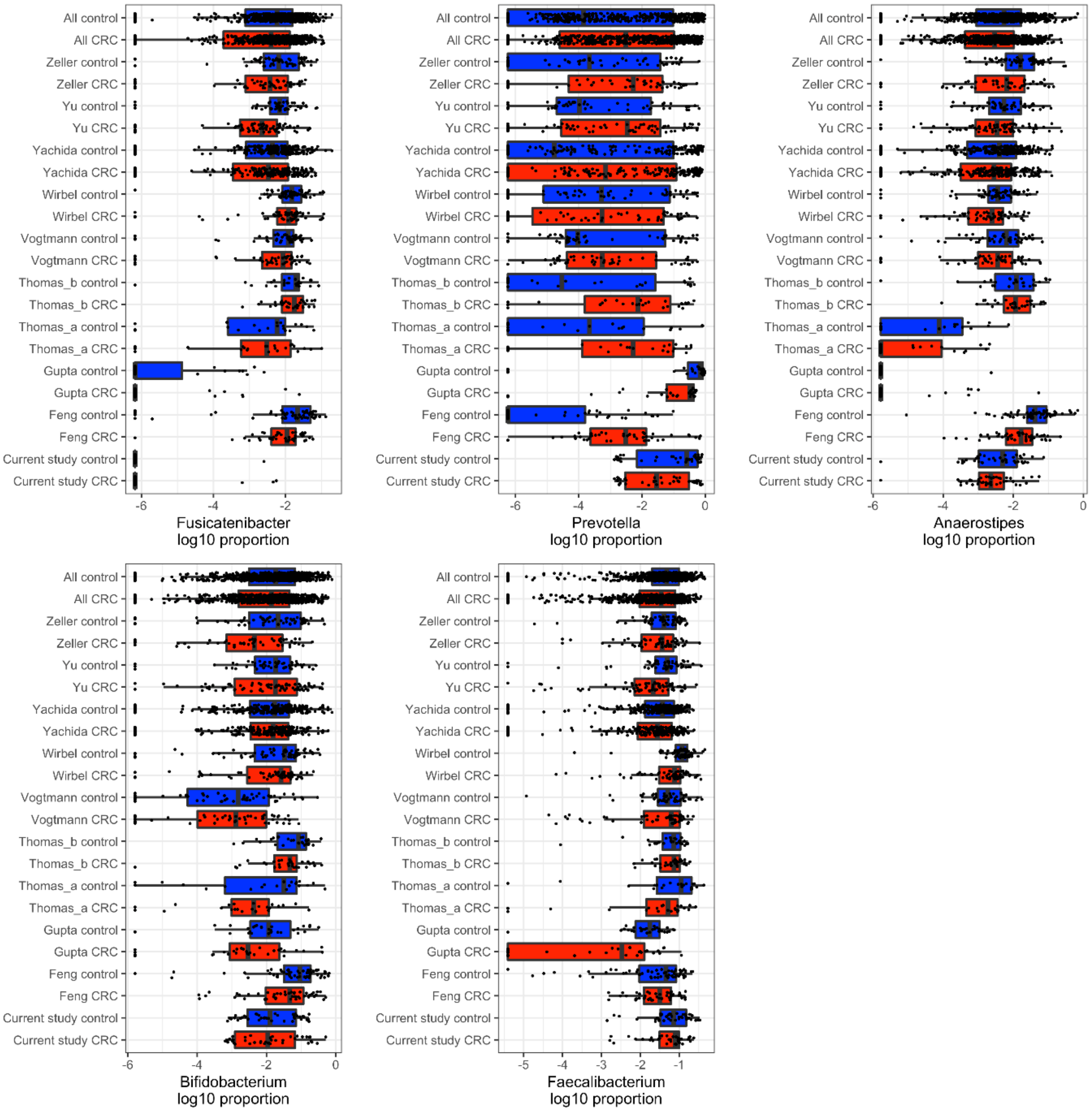

Figure S4A. Distributions of relative abundance of genera of greatest importance to random forest models. The boxplots labelled 'All' are a summary of all of the studies, including the current study. The first 19 taxa are CRC-enriched (mean relative abundance) in the majority of studies; the final 5 taxa are control-enriched (mean relative abundance) in the majority of studies.

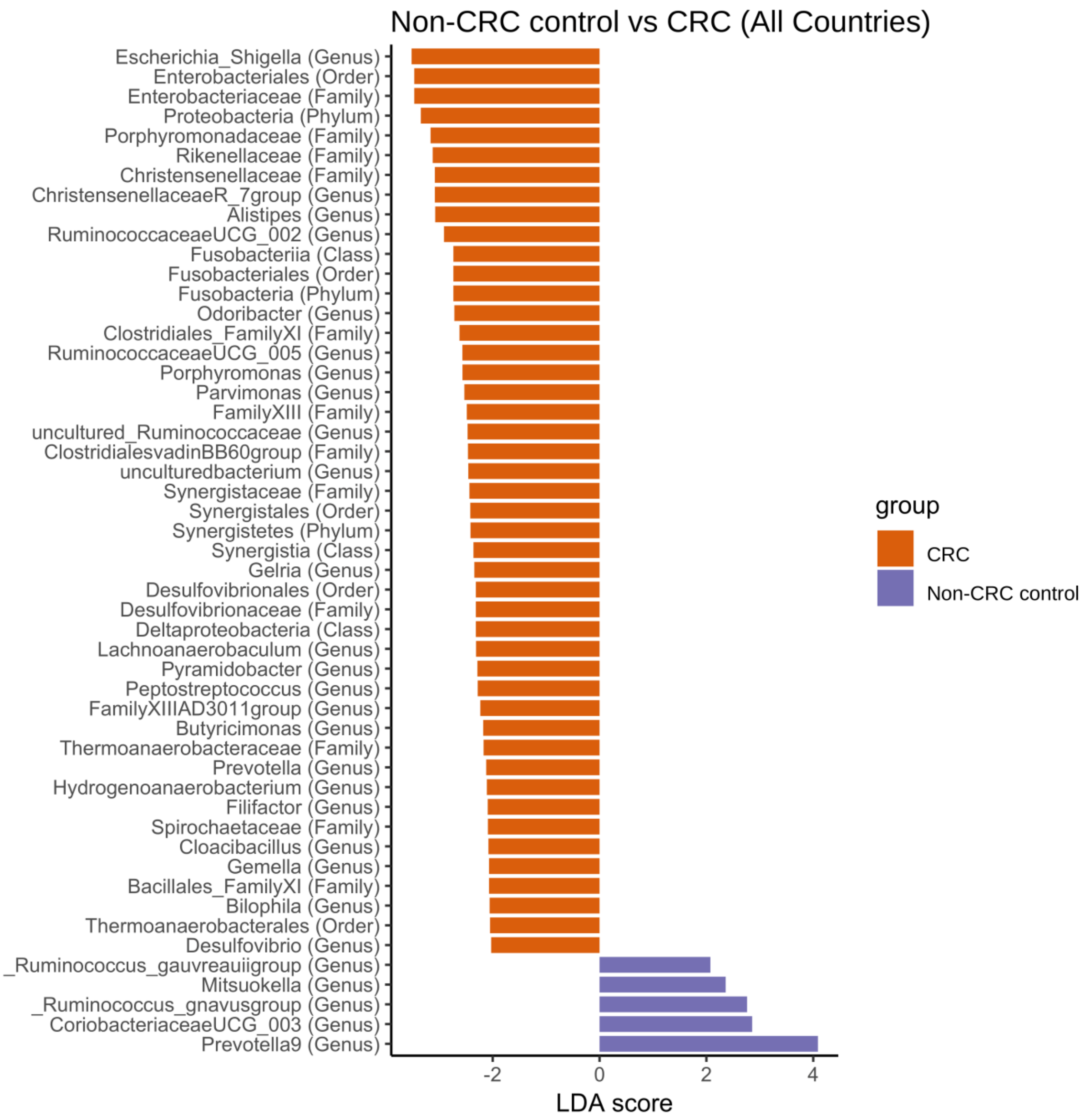

Figure S4B. LEfSe plot illustrating taxa enriched in CRC compared with non-CRC controls for the current study cohort as a whole.

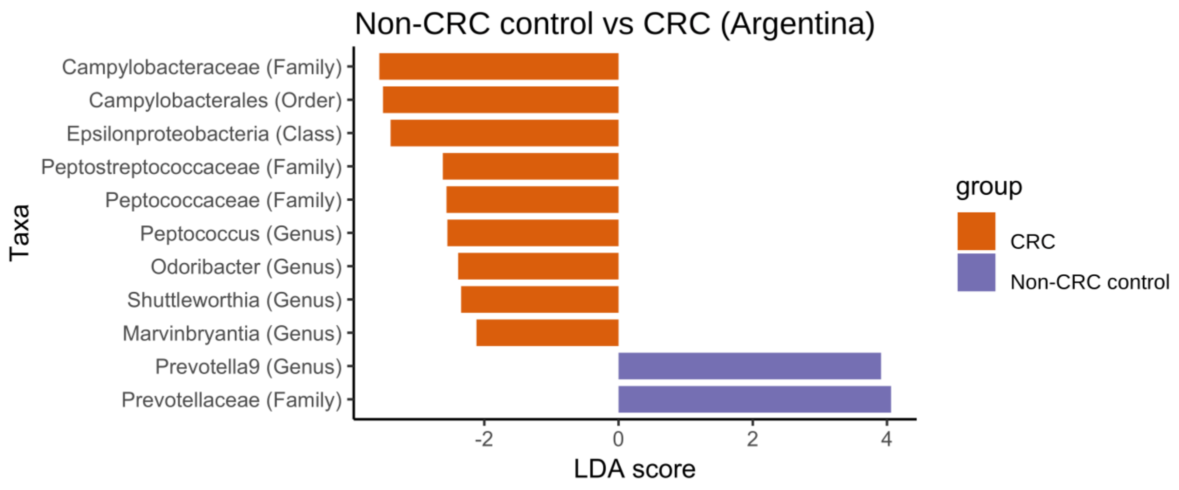

Figure S4C. LEfSe plot illustrating taxa enriched in CRC compared with non-CRC controls (Argentina).

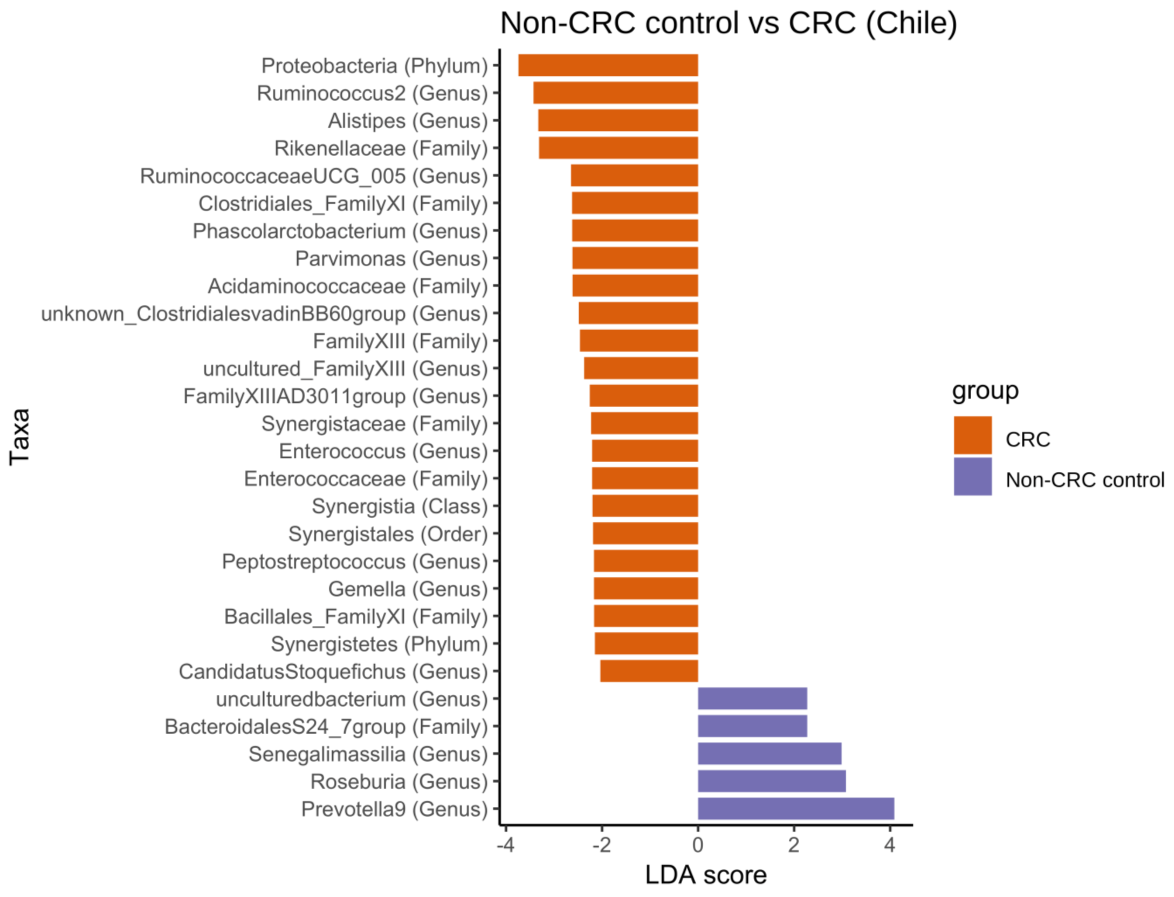

Figure S4D. LEfSe plot illustrating taxa enriched in CRC compared with non-CRC controls (Chile).

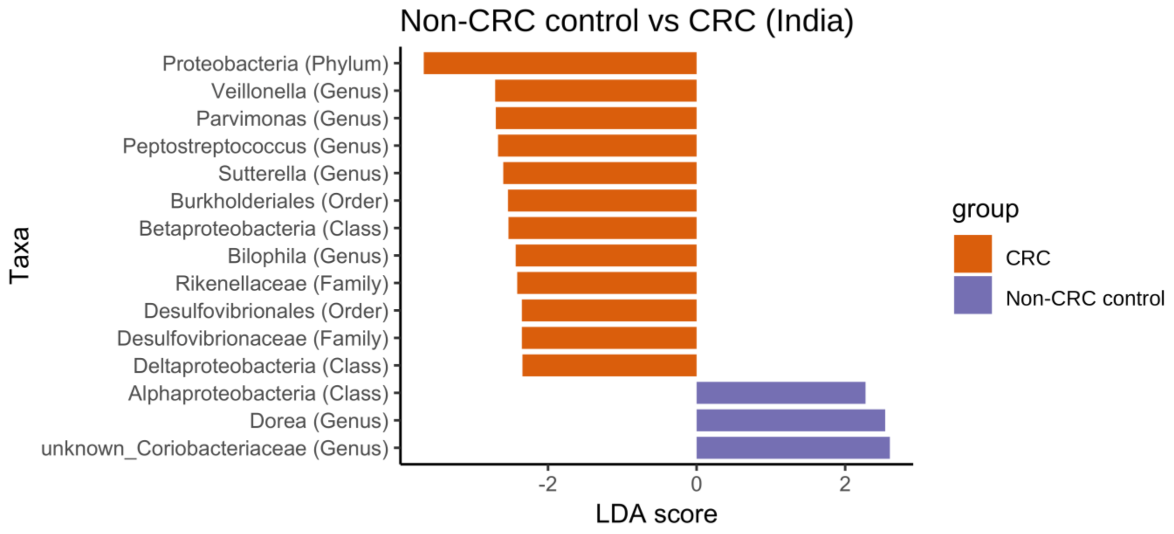

Figure S4E. LEfSe plot illustrating taxa enriched in CRC compared with non-CRC controls (India).

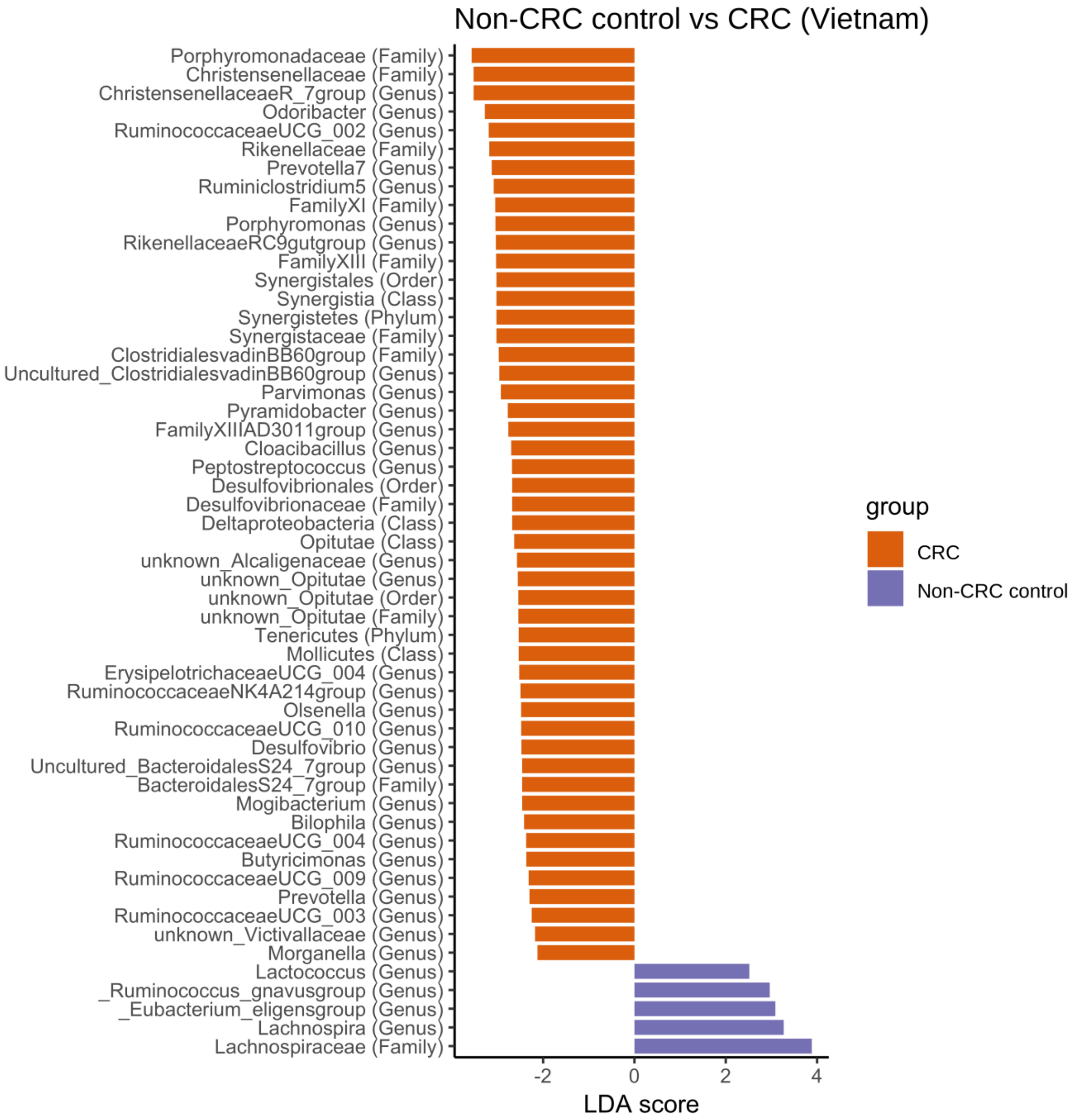

Figure S4F. LEfSe plot illustrating taxa enriched in CRC compared with non-CRC controls (Vietnam).
